# Germacrane Sesquiterpene Dilactones from *Mikania micrantha* and Their Antibacterial and Cytotoxic Activity

**DOI:** 10.3390/molecules28052119

**Published:** 2023-02-24

**Authors:** Li-Mei Dong, Qiao-Lin Xu, Shao-Bo Liu, Shan-Xuan Zhang, Meng-Fei Liu, Jin-Long Duan, Jin-Kui Ouyang, Jia-Tao Hu, Fen-Yu Fu, Jian-Wen Tan

**Affiliations:** 1 State Key Laboratory for Conservation and Utilization of Subtropical Agro-bioresources/Guangdong Key Laboratory for Innovative Development and Utilization of Forest Plant Germplasm, College of Forestry and Landscape Architecture, South China Agricultural University, Guangzhou 510642, China; 2 Guangdong Eco-Engineering Polytechnic, Guangzhou 510520, China; 3 Guangdong Provincial Key Laboratory of Silviculture, Protection and Utilization, Guangdong Academy of Forestry, Guangzhou 510520, China

**Keywords:** *Mikania micrantha*, germacrane sesquiterpene, antibacterial, anti-*MRSA*, cytotoxicity

## Abstract

Four new germacrane sesquiterpene dilactones, 2*β*-hydroxyl-11*β*,13-dihydrodeoxymikanolide (**1**), 3*β*-hydroxyl-11*β*,13-dihydrodeoxymikanolide (**2**), 1*α*,3*β*-dihydroxy-4,9-germacradiene-12,8:15,6-diolide (**3**), and (11*β*,13-dihydrodeoxymikanolide-13-yl)-adenine (**4**), together with five known ones (**5**–**9**) were isolated from the aerial parts of *Mikania micrantha*. Their structures were elucidated on the basis of extensive spectroscopic analysis. Compound **4** is featured with an adenine moiety in the molecule, which is the first nitrogen-containing sesquiterpenoid so far isolated from this plant species. These compounds were evaluated for their in vitro antibacterial activity against four Gram-(+) bacteria of *Staphyloccocus aureus* (*SA*), methicillin-resistant *Staphylococcus aureus* (*MRSA*), *Bacillus cereus* (*BC*) and *Curtobacterium. flaccumfaciens* (*CF*), and three Gram-(–) bacteria of *Escherichia coli* (*EC*), *Salmonella. typhimurium* (*SA*), and *Pseudomonas Solanacearum* (*PS*). Compounds **4** and **7**–**9** were found to show strong in vitro antibacterial activity toward all the tested bacteria with the MIC values ranging from 1.56 to 12.5 µg/mL. Notably, compounds **4** and **9** showed significant antibacterial activity against the drug-resistant bacterium of *MRSA* with MIC value 6.25 µg/mL, which was close to reference compound vancomycin (MIC 3.125 µg/mL). Compounds **4** and **7**–**9** were further revealed to show in vitro cytotoxic activity toward human tumor A549, HepG2, MCF-7, and HeLa cell lines, with IC_50_ values ranging from 8.97 to 27.39 μM. No antibacterial and cytotoxic activity were displayed for the other compounds. The present research provided new data to support that *M. micrantha* is rich in structurally diverse bioactive compounds worthy of further development for pharmaceutical applications and for crop protection in agricultural fields.

## 1. Introduction

*Mikania micrantha* H. B. K. (Asteraceae) is a perennial creeping vine indigenous to Central and South America. However, for the people in some other tropical and subtropical areas of the world, especially in Southeast and South Asia, Australia, and some Pacific islands, this plant is more often recognized by the local people as a harmful exotic invasive plant [1]. In those new Asia-Pacific habitats far away from its native America homeland, *M. micrantha* grows very fast and has been significantly hampering the normal growth of other local plants and thereby seriously threatening the local biodiversity, due to which *M. micrantha* has now been recorded as one of the 100 most invasive species in the world [2]. It was reported that the first appearance of *M. micrantha* in China was in the 1980s and now it has spread widely and rapidly in a large area of southern China; its long-lasting invasion has caused huge economic losses to the local agricultural and forestry production, and seriously damaged the original ecological balance [1,3].

Apart from the recognition of this plant as a very successful exotic invasive plant species, it is very interesting to know that, in Mexico, *M. micrantha* has long been used as a traditional folk remedy for the treatments of skin diseases, snake bites, and scorpion stings [4]. In addition, this plant has long been used in Jamaica as a traditional herbal medicine to treat skin itches and athlete’s foot [5]. In the past twenty years or so, some bioactive screening studies on exploring the potential values of this plant species have indicated that *M. micrantha* possesses a wide range of biological activities, including antibacterial, antitumor, analgesic, cytotoxic, and phytotoxic activities [5,6,7,8,9,10,11,12,13,14]. These literature reports indicated that potentially rich biologically active chemical constituents would certainly exist in this plant. To date, some phytochemical studies conducted by several research groups in different countries have discovered a series of structurally diverse chemicals from this plant species, including terpenoids, steroids, flavonoids, and phenolic compounds, but only a small part of those identified compounds were addressed for their biological activities [11,15,16,17,18,19,20,21,22,23,24].

**Figure 1:**
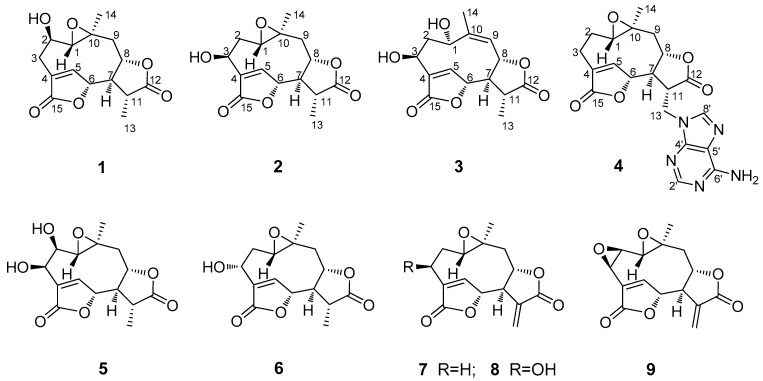
Chemical structures of compounds **1–9**.

Recently, in our pre-test, we noticed that the extracts of the aerial part material of this plant collected in Guangzhou, China, showed obviously detectable antibacterial and cytotoxic activity, and therefore we initiated a phytochemical study on this plant, by which a group of rare C-9 hydroxylated *ent*-kaurene diterpene glucosides and some phenolic compounds with antioxidant activities were revealed [25,26]. During our ongoing effort to clarify those potential antibacterial and cytotoxic natural products in this plant, a series of germacrane sesquiterpene dilactones, including four new (**1**–**4**) and five known ones (**5**–**9**) (Figure 1), were further isolated and identified from the aerial parts of *M. micrantha*. The new structures were established based on detailed analysis of their MS and NMR spectra (see Supplementary Materials). Among those isolates, compound **4** is novelly featured with an adenine moiety in the molecule which is the first nitrogen-containing sesquiterpenoid so far isolated from this plant species. The in vitro bioassays further revealed that part of these germacrane sesquiterpene dilactones showed significant antibacterial (including anti-*MRSA*) and cytotoxic activities. In this paper, we describe the isolation and structure elucidation of these compounds, as well as the evaluation for their in vitro antibacterial and cytotoxic activity.

## 2. Results and Discussion

The air-dried and powdered leaf material of *M. micrantha* was extracted with 95% ethanol (in water). The resultant crude ethanol extract was then suspended with water and sequentially partitioned with petroleum ether (PE), ethyl acetate (EtOAc), and n-butanol (n-But), respectively, to generate the PE, EtOAc, and n-But extracts. The obtained EtOAc extract was then subjected to a series of column chromatographic steps over silica gel, ODS, and Sephadex LH-20 to afford the four new (**1**–**4**) and five known (**5**–**9**) germacrane sesquiterpene dilactones.

**Figure 2:**
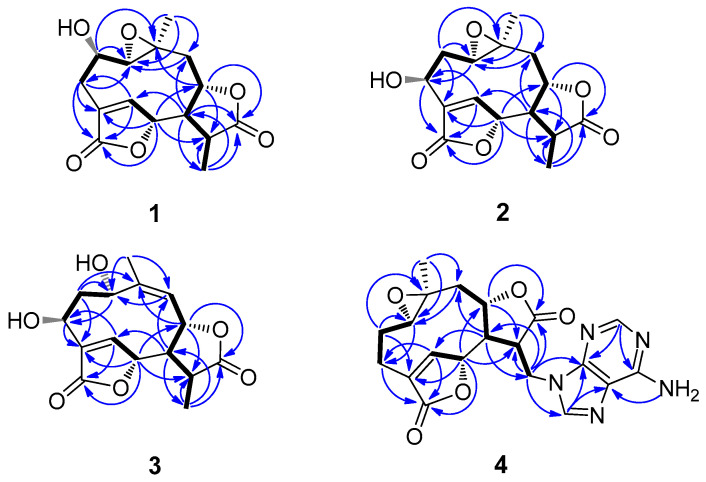
Key ^1^H-^1^H COSY (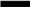) and selected HMBC (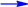) correlations of **1**–**4**.

**Figure 3:**
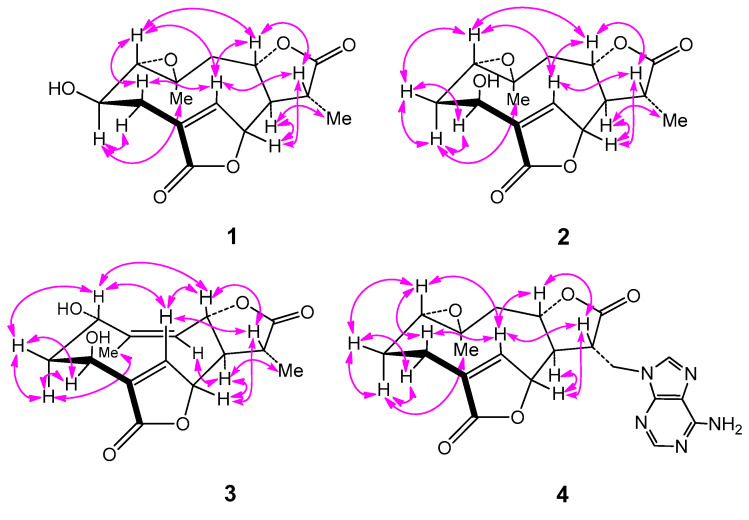
Key NOESY (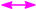) correlations of compounds **1**–**4**.

Compound **1**, obtained as colorless needles, was determined to have a molecular formula C_15_H_18_O_6_ from pseudo-molecular ions at *m*/*z* 317 [M + Na]^+^, 611 [2M + Na]^+^ in positive ESI-MS and *m*/*z* 293 [M − H]^−^ and 329 [M + Cl]^−^ in negative ESI-MS as well as *m*/*z* 329.0805 [M + Cl]^−^ in HR-ESI-MS (calcd for C_15_H_18_O_6_Cl^−^, 329.0797). In the ^1^H NMR spectrum (Table 1), signals for a tertiary methyl group at *δ* 1.40 (3H, d, Me-13), a quaternary methyl group at *δ* 1.25 (3H, s, Me-14), three oxygenated methine groups at *δ* 3.73 (1H, H-2), 5.27 (1H, H-6) and 4.64 (1H, H-8), an oxymethine proton belonging to an epoxy group at 2.85 (1H, H-1) and an olefinic proton at *δ* 7.79 (1H, H-5) were all observed. The ^13^C NMR spectrum (Table 2), coupled with HSQC analysis, showed resonances for fifteen carbons (4×C, 7×CH, 2×CH2, 2×CH3), in which two lactone carboxyl carbons, four oxymethines, one oxygenated quaternary carbon, one olefinic methine, and one olefinic quaternary carbon were deduced. The ^1^H–^1^H COSY spectrum showed correlations of H-1/H-2/H_2_-3, H-5/H-6, and H_2_-9/H-8/H-7/H-11/H_3_-13, indicating the presence of structural fragments of C-1–C-2–C-3, C-5–C-6 and C-9–C-8–C-7–C-11–C-13 (Figure 2). In the HMBC spectrum, the observation of ^1^H−^13^C long-range correlation signals from *δ*_H_ 7.79 (1H, H-5) to 32.2 (C-3) and 174.3 (C-15), and from *δ*_H_ 2.58 (1H, H-3β) to *δ*_C_ 174.3 (C-15) supported the connections of C-4 with C-3, C-5, and C-15. The HMBC correlations from *δ*_H_ 5.27 (1H, H-6) to 79.2 (C-8) and 41.4 (C-11), and from *δ*_H_ 2.62 (1H, H-7) to *δ*_C_ 151.4 (C-5), 79.2 (C-8) and 13.5 (C-13) supported the connections of C-7 with C-6, C-8, and C-11. The HMBC correlations from *δ*_H_ 1.25 (1H, H-14) to 66.9 (C-1), 44.4 (C-9) and 58.8 (C-10), and from *δ*_H_ 3.73 (1H, H-2) to *δ*_C_ 58.8 (C-10) supported the direct linkages of C-10 with C-1, C-9 and C-14. The HMBC correlations from *δ*_H_ 1.40 (1H, H-13) to 178.0 (C-12) supported C-11 linked with the carboxyl carbon C-12. The HMBC correlations from H-6 to C-15 and from *δ*_H_ 4.64 (1H, H-8) to C-12 supported the ester bond linkages of C-6 with C-15 and of C-8 with C-12, respectively. Those above spectral data and literature precedents supported **1** to be a germacrane sesquiterpene dilactone [19,24]. Careful comparison indicated that the ^1^H and ^13^C NMR spectral data of **1** closely resembled those of a previously reported known germacrane sesquiterpenoid 1,10-epoxy-4-germacrene-12,8;15,6-diolide [16], except that the resonances for the methylene group at C-2 in the known compound were absent in **1**. Instead, additional signals for an oxygenated methine group [*δ*_H_ 3.73 (1H, H-2); *δ*_C_ 66.9 (C-2)] were exhibited in the spectra. This observation supported that the structure of **1**, as shown in Figure 1, was close to 1,10-epoxy-4-germacrene-12,8;15,6-diolide with the only difference that one more hydroxy group attached at C-2 in **1**. This deduction was in complete accord with the molecular formula of **1** and the *β*-orientation of the hydroxy group at C-2 was supported by the observed large coupling constant of H-1 with H-2 (*J*_H-1*β*,H-2*α*_ = 11.7 Hz) [19]. Moreover, the *β*-orientation of the 2-hydroxy group was further well evidenced by the NOESY spectrum, in which significant NOE correlation signals of H-2 with Me-14, but not H-2 with H-1*β*, were observed (Figure 3). Therefore, the structure of compound **1** was identified as 2*β*-hydroxyl-11*β*,13-dihydrodeoxymikanolide, trivially named dihydrodeoxymikanolide M.

Compound **2** was deduced to have a molecular formula C_15_H_18_O_6_, the same as that of compound **1**, as determined by HR-ESI-MS (*m*/*z* 329.0792 [M + Cl]^−^, calcd for C_15_H_18_O_6_Cl^−^, 329.0797). The ^1^H NMR spectrum (Table 1) of **2** showed signals readily recognized for two methyl groups at *δ*_H_ 1.40 (3H, d, Me-13) and 1.19 (3H, s, Me-14), one olefinic methine at *δ*_H_ 7.81 (1H, H-5), and four oxymethines at *δ*_H_ 3.07 (1H, dd, H-1), 4.88 (1H, dd, H-3), 5.33 (1H, H-6) and 4.57 (1H, ddd, H-8). The ^13^C NMR data (Table 1) supported the above analysis, which showed fifteen carbon signals including two methyls at *δ*_C_ 13.5 (C-13) and 20.6 (C-14), two methylenes, seven methines (including one olefinic methine at *δ*_C_ 147.8 (C-5) and four oxygenated methines at *δ*_C_ 60.2 (C-1), 65.8 (C-3), 81.8 (C-6), and 79.5 (C-8)), one oxygenated quaternary carbon at *δ*_C_ 58.5 (C-10), and two carboxyl carbon at *δ*_C_ 178.1 (C-12) and 172.6 (C-15). The closely resembled NMR spectral data (Table 1 and Table 2) of **2** with those of **1** supported **2** also being a germacrane sesquiterpene dilactone close to **1**. Detailed comparison of the ^1^H- and ^13^C-NMR spectra revealed that the signals for the hydroxy-methine group at C-2 and the methylene group at C-3 in **1** were replaced by signals for a new methylene group (*δ*_H_ 1.75 (1H, ddd, H-2*α*), 2.17 (1H, ddd, H-2*β*); *δ*_C_ 32.8 (C-2)) and a new oxymethine group (*δ*_H_ 4.88 (1H, dd, H-3); *δ*_C_ 65.8 (C-3)), respectively. This observation indicated the difference between the two compounds that the hydroxy group located at C-2 in **1** was changed to attach at C-3 in **2**. The rationality for assigning the hydroxy group at C-3 in **2** was supported by observation of significant HMBC correlations from *δ*_H_ 7.81 (1H, H-5) to *δ*_C_ 65.8 (C-3) (Figure 3), and accurately interpreted the significant chemical shift change of C-4 and C-5 from *δ*_C_ 131.1 (C-4) and 151.4 (C-5) in **1** to *δ*_C_ 138.0 (C-4) and 147.8 (C-5) in **2**, respectively. Furthermore, the *β*-orientation of the hydroxy group at C-3 was supported by the observed small coupling constants of H-3 with H-2 (*J*_H-3*α*,H-2*α*_ = 4.0 Hz and *J*_H-3*α*,H-2*β*_ = 2.2 Hz) [19] and further well evidenced by the NOESY spectrum, in which significant NOE correlations of H-3 with H_2_-2, but no NOE correlation of H-3 with H-1, were displayed (Figure 3). Moreover, compound **2** showed closely similar chemical shift values and proton coupling constants from C-6 through C-14 (Table 1 and Table 2) to those of **1**, which indicated that the other part structure and relative configurations (from C-6 through C-14) of **2** was the same as that of **1**. Thus, compound **2** was identified as 3*β*-hydroxyl-11*β*,13-dihydrodeoxymikanolide, trivially named dihydrodeoxymikanolide N, and all the other spectroscopic data supported this deduction.

Compound **3** was obtained as a colorless oil with a molecular formula C_15_H_18_O_6_ the same as that of compound **2**, as determined by HR-ESI-MS, *m*/*z* 329.0802 [M + Cl]^−^ (calcd. for C_15_H_18_O_6_Cl^−^, 329.0797). The closely related ^1^H- and ^13^C-NMR (Table 1) spectral data of **3** with those of **2** supported **3** being an isomeric germacrane sesquiterpenoid of **2**. Comparison of the NMR spectral data revealed the major difference between the two compounds, that the resonances in **2** for the oxygenated quaternary carbon at C-10 and for the methylene group at C-9 were absent in **3**. Instead, additional signals for one olefinic quaternary carbon (*δ*_C_ 148.2 (C-10)) and one olefinic methine group (*δ*_C_ 121.8 (C-9) and *δ*_H_ 5.29 (1H, d, H-9)) were exhibited in the spectra. This observation led to primarily establish **3**, as shown in Figure 1, to be a compound possessed a similar structural skeleton to compound **2**, with the only difference of the epoxy group at C-1(C-10) and the methylene group at C-9 in **2** was replaced by a hydroxy-methine group at C-1 and a trisubstituted double bond between C-9 and C-10, respectively. This deduction was consistent with the molecular formula of **3** and further well supported by the following 1D and 2D-NMR spectral analysis. In the ^1^H–^1^H COSY spectrum, coupled with HSQC analysis, correlation signals for H-atom spin systems corresponding to structural fragments of C-1–C-2–C-3, C-5–C-6 and C-9–C-8–C-7–C-11–C-13 (Figure 2) were all displayed. The HMBC spectrum showed significant ^1^H−^13^C long-range correlations from *δ*_H_ 8.12 (1H, H-5) to *δ*_C_ 65.7 (C-3), 138.6 (C-4) and 171.8 (C-15), and from *δ*_H_ 5.47 (1H, H-6) to C-4 and C-15, which confirmed the connections of C-4 with C-3, C-5 and C-15, and evidenced the ester bond linkage between C-6 and C-15. The observation of HMBC correlations from *δ*_H_ 2.62 (1H, H-7) to *δ*_C_ 145.6 (C-5), 13.2 (C-13), from *δ*_H_ 5.47 (1H, H-6) to 38.7 (C-11), and from *δ*_H_ 1.38 (H-13) to *δ*_C_ 51.8 (C-7), 177.5 (C-12) and 38.7 (C-11), evidenced the connections of C-7 with C-6, and C-11 with C-7, C-12 and C-13. The HMBC correlations from *δ*_H_ 2.09 (Me-14) to *δ*_C_ 64.4 (C-1), 121.8 (C-9), 148.2 (C-10) supported C-10 linked with C-1, C-9, and C-14. The HMBC correlation from H-8 to C-12 supported the ester bond linkage of C-8 with C-12. Furthermore, both the *α*-orientation of the hydroxy group at C-1 and the *β*-orientation of the hydroxy group at C-3 were first supported by the large coupling constant of H-1 with H-2*α* (*J*_H-1*β*,H-2*α*_ = 9.3 Hz) and the small coupling constant of H-3*α* with H-2*α* (*J*_H-3*α*,H-2*α*_ = 3.3 Hz) [19], and further supported by NOESY experiment, in which H-1 showed significant NOE correlations with H-5 and H-8, while no NOE correlations of H-1 with H-2*α* and H-3*α* (Figure 3). Consequently, the structure of compound **3**, as shown in Figure 1, was elucidated as 1*α*,3*β*-dihydroxy-4,9-germacradiene-12,8:15,6-diolide, trivially named dihydrogermacradienolide.

Compound **4** was obtained as a white powder. Its molecular formula C_20_H_21_N_5_O_5_ was determined by HR-ESI-MS (*m*/*z* 446.1248 [M + Cl]^−^, calcd for C_20_H_21_N_5_O_5_Cl^−^, 446.1237). The presence of an adenine moiety in the molecule was suggested by the presence of characteristic proton signals at *δ*_H_ 8.14 (1H, s, H-2′) and 8.06 (1H, s, H-8′) in the ^1^H NMR spectrum, and the carbon signals in the ^13^C NMR spectrum at *δ*_C_ 152.6 (C-2′), 149.7 (C-4′), 118.6 (C-5′), 156.0 (C-6′) and 141.1 (C-8′) [27]. Apart from those signals due to the adenine moiety, the remaining signals in the ^1^H NMR spectrum for the aglycone of **2** were recognizable for a methyl group at *δ*_H_ 0.98 (3H, Me-14), an olefinic methine at *δ*_H_ 7.70 (1H, H-5), two oxymethines at *δ*_H_ 4.93 (1H, H-6) and 4.60 (1H, m, H-8), and one oxymethine proton belonging to an epoxy group at *δ*_H_ 2.77 (1H, dd, H-1). The ^13^C NMR spectrum indicated, beside the signals for the adenine moiety, fifteen carbons including one methyl, four methylenes, six methines (including an olefinic methine at *δ*_C_ 149.4 (C-5) and three oxygenated methines at *δ*_C_ 60.9 (C-1), 79.9 (C-6) and 77.5 (C-8)), and four quaternary carbons (including an olefinic quaternary carbon at *δ*_C_ 131.1 (C-4) and two carboxyl carbons at *δ*_C_ 173.8 (C-12), 171.9 (C-15)). By comparison, it was found that the ^1^H- and ^13^C-NMR spectroscopic data (Table 1 and Table 2) of the aglycone of **4** were quite similar to those of the known compound 1,10-epoxy-4-germacrene-12,8;15,6-diolide [16], except that the resonances for the methyl group at C-13 in the known compound were replaced by the signals for a methylene group (*δ*_C_ 41.8 (C-13) and *δ*_H_ 4.64 (1H, m, H-13a), 4.50 (1H, dd, H-13b)) in **4**. These findings supported the structure of **4**, as shown in Figure 1, being a closely related derivative of 1,10-epoxy-4-germacrene-12,8;15,6-diolide with specially an adenine moiety linked at C-13. This deduction was in complete accord with the molecular formula of **4**, and the linkage of the adenine moiety at C-13 was well supported by the observation of significant ^1^H–^13^C long-range correlations of H_2_-13 (*δ*_H_ 4.64, 4.50) with *δ*_C_ 149.7 (C-4′) and 141.1 (C-8′) in the HMBC spectrum. Moreover, the ^1^H–^1^H COSY and HMBC spectra showed significant correlation signals permitting to establish the structural fragments of C-1–C-2–C-3, C-5–C-6 and C-9–C-8–C-7–C-11–C-13, and construct their connections as depicted in Figure 3. The same relative configurations of the aglycone part of **4** as those of 1,10-epoxy-4-germacrene-12,8;15,6-diolide were well confirmed by the NOESY spectrum which displayed significant NOE correlation signals as delineated in Figure 3. Therefore, the structure of compound **4** was unambiguously identified as (11*β*,13-dihydrodeoxymikanolide-13-yl)-adenine, trivially named dihydrodeoxymikadenine, as shown in Figure 1.

The five known compounds obtained in this study were identified as 2*β*,3*β*-dihydroxy-11*β*,13-dihydroxydeoxymikanolide (**5**) [19], 3*α*-hydroxy-11*β*,13-dihydroxydeoxymikanolide (**6**) [19], deoxymikanolide (**7**) [16], 3*β*-hydroxy-deoxymikanolide (**8**), and mikanolide (**9**) [28] by comparison of their spectroscopic data with literature values.

All nine germacrane sesquiterpenoids were evaluated for their in vitro antibacterial activity against four Gram-(+) bacteria of *Staphyloccocus aureus* (*SA*), methicillin-resistant *Staphylococcus aureus* (*MRSA*), *Bacillus cereus* (*BC*), and *Curtobacterium flaccumfaciens* (*CF*), and three Gram-(−) bacteria of *Escherichia coli* (*EC*), *Salmonella typhimurium* (*ST*), and *Pseudomonas solanacearum* (*PS*), by using a microdilution titer method as described in the experimental section [29]. As shown in Table 3, compounds **4** and **7**–**9** were found to display significant in vitro antibacterial activity toward all the tested bacteria with MIC values ranging from 6.25 to 12.5 µg/mL, which were close to that of the reference compounds kanamycin sulfate and vancomycin, while no detectable antibacterial activity was displayed for the other compounds. Among the seven test microorganisms, *SA*, *BC*, *EC,* and *ST* were human pathogenic bacteria, and *CF* and *PS* were two Agro-pathogenic bacteria capable of widely causing crop infection diseases. The exhibition of strong and broad-spectrum antibacterial activity of compounds **4** and **7**–**9** against all the test human pathogenic and agro-pathogenic bacteria in this bioassay indicated that these four compounds were not only valuable to be developed in medical usage as antibacterial agents for the treatment of human infectious diseases, but also valuable to be applied in agriculture for crop protection. It is worth noting that compounds **4** and **7**–**9** showed significant antibacterial activity against *MRSA*. *MRSA* infection is responsible for a rapidly increasing number of serious infectious diseases severely threatening global public health [30,31], and its control and clinic therapy are urgently lacking effective and safe anti-*MRSA* agents. The strong anti-*MRSA* activity of these four compounds suggested that they have the potential to be developed as an effective anti-*MRSA* agent.

Compounds **1**–**9** were further screened for their in vitro cytotoxic activity against human tumor A549, HepG2, MCF-7, and HeLa cell lines, using a microdilution titer technique as recently we described [32]. The resulting IC_50_ values are displayed in Table 4, compared to Adriamycin as positive control. Compounds **4** and **7**–**9** were found to obviously show cytotoxic activity against all the four tested cancer cell lines, with IC_50_ values ranging from 8.97 to 27.39 μM. No obvious cytotoxic activity was displayed for the other compounds. The bioassay data indicated that compounds **4** and **7**–**9** were bioactive compounds with certain anticancer potentials highly valuable to be further developed as therapeutic drugs or health care agents for cancer treatment or prevention.

Based on comparison of the chemical structures and their antibacterial and cytotoxic activities of compounds **1**–**6** (except **4**) versus **7**–**9**, we can find out that the existence of the *α*-methylene-*γ*-lactone moiety, i.e., the carboxyl group of O=C(12) conjugated with the exocyclic double bond –C(11)=C(13)H_2_, would be essential for this type of germacrane sesquiterpenoid to display strong antibacterial potential. It was reported that the mechanism for those sesquiterpenoids, characterized with a *α*-methylene-*γ*-lactone moiety in the molecule, to display strong antibacterial and cytotoxic activity, it would be due to the potential Michael-type addition reactions that easily happen in vivo by nucleophiles, such as thiol groups of cysteine residues in proteins, to react with the *α*-methylene-*γ*-lactone moiety [33]. However, at this point, it was exceptional for compound **4** which also showed strong antibacterial and cytotoxic activity toward all the seven bacterial strains, but evidently, this compound did not contain the aforementioned *α*-methylene-*γ*-lactone moiety in the structure. In fact, compound **4** was novelly characterized with a nitrogen-containing adenine motif attaching at C-13 in the molecule, which suggested that compound **4** might employ a different mechanism to show its antibacterial and cytotoxic activity, and the present data supported a presumption that the introduction of nitrogen atom or nitrogen-containing moiety into the structure might be another effective way to remarkably enhance the antibacterial and cytotoxic potentials of sesquiterpenoids, at least reasonable for this type of sesquiterpene dilactones [34].

As a very successful invasive plant, *M. micrantha* can annually produce a huge plant biomass at its invasion areas, but this biomass resource has yet not been well-developed and utilized so far. In recent years, some phytochemical studies on *M. micrantha* have revealed a variety of chemical constituents, with some of them addressed with biological activities, and those findings also indicated the existence of variability in the chemical constituent compositions among various populations of this plant species distributed in different parts of the world. In the present study, the obtained data provided new evidences to supported that the populations of *M. micrantha* growth in Guangzhou area in China is rich in bioactive natural products, at least rich in antibacterial and cytotoxic chemicals, potentially highly valuable to be developed in medical usage or in agriculture for crop protection.

## 3. Materials and Methods

### 3.1. General Experimental Procedures

Optical rotations were measured by a Perkin-Elmer 341 polarimeter (Perkin-Elmer, Inc., Waltham, MA, USA). UV spectra were acquired on a Perkin-Elmer Lambda 650 UV-Vis spectrometer (Perkin-Elmer, Inc.,Waltham, MA, USA). General electrospray ionization-mass spectrometry (ESI-MS) were recorded on a *MDS SCIEX API 2000* liquid chromatography/tandem mass spectrometry (LC/MS/MS) instrument (Applied Biosystems, Inc., Forster, CA, USA). High-resolution ESI-MS (HR-ESI-MS) were acquired on a *Bruker maXis* spectrometer (Bruker Daltonics GmbH, Bremen, Germany). Nuclear magnetic resonance (NMR) spectra were carried out on a *Bruker AVANCE HD 500* spectrometer (Bruker Biospin Gmbh, Rheistetten, Germany) with deuterated solvent residues as internal standard. Silica gel (80–100 and 200–300 mesh, Qingdao Haiyang Chemical Co., Qingdao, China), YMC ODS-A (50 μm, YMC Co., Ltd., Kyoto, Japan) and Sephadex LH-20 (Pharmacia Fine Chemcial Co. Ltd., Uppsala, Sweden) were used for open column chromatography (CC). ODS CC was carried out on a CXTH P3000 instrument (Beijing Chuang Xin Tong Heng Science and Technology Co., Ltd., Beijing, China) equipped with a UV 3000 UV–vis Detector and a C-18 column (50 μm, 50 × 500 mm). Preparative HPLC was performed with an HPLC system equipped with a Shimadzu LC-6AD pump and a Shimadzu RID-10A refractive index detector using a Shim-pack PRC-ODS C-18 column (5 μm, 20 × 250 mm). Fractions were monitored by precoated HSGF_254_ TLC (Yantai Jiangyou Silica Gel Development Co., Ltd., Yantai, China) and spot detection was performed by spraying 10% H_2_SO_4_ in ethanol, followed by heating. Kanamycin sulfate, resazurin, vancomycin, and adriamycin were purchased from Sigma-Aldrich (Shanghai, China) Trading Co. (Shanghai, China).

### 3.2. Plant Material

The aerial part materials of *M. micrantha* was collected at the South China Botanical Garden, Guangzhou, Guangdong Province, P.R. China, and identified by Dr. Xinsheng Qin at the College of Forestry and Landscape Architecture, South China Agricultural University. A voucher specimen (No.20190623) was deposited in the phytochemical laboratory at the College of Forestry and Landscape Architecture, South China Agricultural University.

### 3.3. Extraction and Isolation

The air-dried aerial part materials of *M. micrantha* (18.5 kg) were powdered and extracted three times with 95% EtOH at room temperature for 3 days each time. After evaporation to remove the solvent ethanol under reduced pressure, the produced viscous concentrate was suspended in water (3 L) and sequentially partitioned with petroleum ether (3 L × 3) and EtOAc (3 L × 3) to afford the corresponding petroleum ether-soluble extract (520 g) and the EtOAc-soluble fraction extract (260 g). The EtOAc-soluble extract was subjected to silica gel column chromatography (CC), eluted with an increasing polarity gradient solvent system of CHCl_3_/MeOH (from 98:2 to 70:30, *v*/*v*, each 15 L) to afford fractions Fr._1_–Fr._8_ after pooling according to their TLC profiles. The fraction Fr._2_ (11 g), collected from the elution of 98:2 CHCl_3_/MeOH, was separated by an ODS CC eluted with a declining polarity solvent system of MeOH/H_2_O (40:60–100:0, *v*/*v*) to provide fractions Fr._2-1_–Fr._2-20_. Fraction Fr._2-10_ was then separated by a silica-gel CC, with elution system of CHCl_3_/Acetone (from 100:0 to 70:30, *v*/*v*), and further purified by a Sephadex LH-20 CC eluted with CHCl_3_/MeOH (1:4, *v*/*v*) to afford compounds **2** (9 mg), **7** (16 mg) and **9** (5 mg). The fraction Fr._3_ (20 g), obtained from the elution of 97:3 CHCl_3_/MeOH, was separated by an ODS CC using a gradient elution system of MeOH/H_2_O (from 40:60–100:0, *v*/*v*) to provide fractions Fr._3-1_–Fr._3-20_. Fraction Fr._3-1_ was first separated by a silica-gel CC, eluted with a gradient solvent system of CHCl_3_/Acetone (from 95:5 to 70:30, *v*/*v*), and then further purified by a Sephadex LH-20 CC with the elution of CHCl_3_/MeOH (1:4, *v*/*v*) to afford **6** (12 mg) and **5** (13.6 mg). The fraction Fr._4_ (18.8 g), obtained from the elution of 95:5 CHCl_3_/MeOH, was separated by an ODS CC using a declining polarity elution system of MeOH/H_2_O (30:70–100:0, *v*/*v*) to give fractions Fr._4-1_–Fr._4-18_. Fraction Fr._4-4_, obtained from the elution with MeOH/H_2_O (40:60), was subjected to a Sephadex LH-20 CC eluted with CHCl_3_/MeOH (1:4, *v*/*v*) to provide pure compound **1** (5 mg). Fraction Fr._4-5_, obtained from the elution of MeOH/H_2_O (45:55), was first subjected to a Sephadex LH-20 CC eluted with pure MeOH and further separated by preparative HPLC with a Shim-pack PRC-ODS C-18 column (5 μm, 20 × 250 mm) using 25% methanol in water (*v*/*v*) as a mobile phase at the flow rate of 10 mL/min to obtain **8** (4 mg, *t*_R_ 105 min). Fraction Fr._4-8_, obtained from the elution of MeOH/H_2_O (55:45), was first subjected to a Sephadex LH-20 CC eluted with CHCl_3_/MeOH (1:4, *v*/*v*) and further separated by preparative HPLC with a Shim-pack PRC-ODS C-18 column (5 μm, 20 × 250 mm) using 10% methanol in water (*v*/*v*) as a mobile phase at the flow rate of 8 mL/min to afford **3** (5 mg, *t*_R_ 70 min). The fraction Fr._8_ (11.3 g), obtained from the elution of 80:20 CHCl_3_/MeOH, was separated by an ODS CC using a gradient elution system of MeOH/H_2_O (from 10:90–100:0, *v*/*v*) to provide fractions Fr._8-1_–Fr._8-17_. Fraction Fr._8-4_, obtained from the elution of MeOH/H_2_O (25:75), was separated by a Sephadex LH-20 CC eluted with CHCl_3_/MeOH (1:4, *v*/*v*) to afford **4** (5 mg).

### 3.4. Spectroscopic Data of Compounds 1–4

*2β-Hydroxyl-11β,13-dihydrodeoxymikanolide* (**1**). Colorless needles. [*α*]${}_{D}^{20}$ +68.0 (*c* 0.50, CH_3_OH); UV (CH_3_OH) λ_max_ nm (log ε) 220 (3.13); CD (CH_3_OH) λ_max_ nm (∆*ε*) 221 (-2.88), 242 (+1.32); ESI-MS (neg.) *m*/*z* 293 [M − H]^−^, 329 [M + Cl]^−^; ESI-MS (pos.) *m*/*z* 317 [M + Na]^+^, *m*/*z* 611 [2M + Na]^+^; HR-ESI-MS (neg.), *m*/*z* 329.0805 [M + Cl]^−^ (calcd for C_15_H_18_O_6_Cl^−^, 329.0797). The ^1^H (500 MHz) and ^13^C (125 MHz) NMR data in methanol-*d*_4_, see Table 1 and Table 2.

*3β-Hydroxyl-11β,13-dihydrodeoxymikanolide* (**2**). Colorless needles. [*α*${]}_{D}^{20}$ +66.9 (*c* 0.63, CH_3_OH). UV (CH_3_OH) *λ*_max_ nm (log *ε*) 220 (3.09). ESI-MS (neg.) *m*/*z* 293 [M − H]^−^, 329 [M + Cl]^−^; ESI-MS (pos.) *m*/*z* 317 [M + Na]^+^, *m*/*z* 611 [2M + Na]^+^. HR-ESI-MS (neg.), *m*/*z* 329.0792 [M + Cl]^−^ (calcd for C_15_H_18_O_6_Cl^−^, 329.0797). The ^1^H (500 MHz) and ^13^C (125 MHz) NMR data in methanol-*d*_4_, see Table 1 and Table 2.

*1α,3β-Dihydroxy-4,9-germacradiene-12,8:15,6-diolide* (**3**). Colorless oil. [*α*]${}_{D}^{20}$ +108.7 (*c* 0.46, C_5_H_5_N). UV (C_5_H_5_N) λ_max_ nm (log ε) 251 (4.11), 257 (4.14). ESI-MS (neg.) *m*/*z* 329 [M + Cl]^−^, 623 [2M + Cl]^−^; ESI-MS (pos.) *m*/*z* 317 [M + Na]^+^, *m*/*z* 611 [2M + Na]^+^. HR-ESI-MS (neg.), *m*/*z* 329.0802 [M + Cl]^−^ (calcd for C_15_H_18_O_6_Cl^−^, 329.0797). The ^1^H (500 MHz, C_5_D_5_N) and ^13^C (125 MHz) NMR data in C_5_D_5_N, see Table 1 and Table 2.

*(11β,13-Dihydrodeoxymikanolide-13-yl)-adenine* (**4**). White amorphous powder. [*α*]${}_{D}^{20}$ +10.5 (*c* 0.60, C_5_H_5_N). UV (C_5_H_5_N) λ_max_ nm (log ε) 260 (3.03). ESI-MS (neg.) *m*/*z* 410 [M − H]^−^, 446 [M + Cl]^−^; ESI-MS (pos.) *m*/*z* 434 [M + Na]^+^, *m*/*z* 845 [2M + Na]^+^. HR-ESI-MS (neg.), *m*/*z* 446.1248 [M + Cl]^−^ (calcd for C_20_H_21_N_5_O_5_Cl^−^, 446.1237). The ^1^H (500 MHz) and ^13^C (125 MHz) NMR data in DMSO-*d*_6_, see Table 1 and Table 2.

### 3.5. Antibacterial Assay

The antibacterial activity of the nine germacrane sesquiterpenoids **1**–**9** was evaluated by using a method as we described recently [29], with slight modifications. In brief, 100 μL indicator solution (resazurin in sterile water, 100 μg/mL) was first placed into each of the sterility control wells (11th column) on 96 well plates, and the indicator solution (about 7.5 mL, 100 μg/mL) was mixed with test bacteria (5 mL, 10^6^ cfu/mL, OD_600_ = 0.07) followed by transferring (100 μL, each) to growth control wells (12th column) and all test wells (1–10th column). Then, each of 100 μL of test compounds (0.8 mg/mL) in beef extract peptone medium, along with the positive control solutions (prepared by kanamycin sulfate and vancomycin instead of the test compounds) and the negative control solution (3% DMSO in beef extract peptone medium), were applied to the wells in the 1st column of the plates. Once all samples (test compounds) and controls were properly applied to the 1st column of wells on the plate, half of the homogenized content (100 μL solution) was then parallel transferred from the 1st column wells to the 2nd column wells, and each subsequent well was treated similarly (doubling dilution) up to the 10th column, followed by discarding the last 100 μL aliquot. Finally, the plates were incubated at 37 °C for about 5–6 h until the color of growth control change to pink. The lowest concentration for each test compound at which color change occurred was recorded as the MIC value of the test compound. In the bioassay, a total of seven microorganisms including four Gram**-**(+) bacteria of *Staphyloccocus aureus* (*SA*), methicillin-resistant *Staphylococcus aureus* (*MRSA*), *Bacillus cereus* (*BC*), *Curtobacterium flaccumfaciens* (*CF*), and three Gram-(–) bacteria of *Escherichia coli* (*EC*), *Salmonella typhimurium* (*SA*), and *Pseudomonas solanacearum* (*PS*) were used in the bioassay.

### 3.6. Cytotoxic Assay

The cytotoxic activity of sesquiterpenoid compounds **1**–**9** against human tumor A549, HepG2, MCF-7, and HeLa cell lines were assayed by using 96 well plates according to a literature MTT method with slight modification [32]. Briefly, the cells for the test were cultured in RPMI-1640 medium supplemented with 10% fetal bovine serum in a humidified atmosphere with 5% CO_2_ at 37 °C. Each well of 96-well cell culture plates was seeded with 100 μL adherent cells (5 × 10^4^ cell/mL) and placed in an atmosphere with 5% CO_2_ at 37 °C for 24 h to form a monolayer on the flat bottoms. Subsequently, the supernatant in each well was removed and 100 μL fresh medium and 100 μL medium containing one of the test compounds was added. Then, the test plate was incubated in a humidified atmosphere with 5% CO_2_ at 37 °C for three days. Afterwards, 20 μL MTT at concentration 5 mg/mL in DMSO was added into each well and incubated for 4 h. Carefully, the supernatant in each well was removed and 150 μL DMSO was added. Then, the plate was vortex shaken for 15 min to dissolve blue formazan crystals. The OD (optical density) value of each well was tested on a Genois microplate reader (Tecan GENios, Männedorf, Switzerland) at 570 nm. All the tests were carried out by three individual experiments and adriamycin was applied as a positive control. In a test, for each of the tumor cell lines, each of the test compounds was set at concentrations 50, 25, 12.5, 6.25, 3.125, and 1.5625 µg/mL. The inhibitory rate of tumor cell growth was calculated by the formula: Inhibition rate (%) = (OD_control_ − OD_treated_)/OD_control_ × 100%, and the IC_50_ values were calculated by SPSS 16.0 statistic software. The four tumor cell lines were obtained from the Kunming Institute of Zoology, CAS. The resulting IC_50_ values listed in Table 4 were based on three individual experiments and represented as means ± standard deviation (SD).

## 4. Conclusions

Four new germacrane sesquiterpene dilactones, trivially named dihydrodeoxymikanolide M (**1**), dihydrodeoxymikanolide N (**2**), dihydrogermacradienolide (**3**) and dihydrodeoxymikadenine (4), along with five known ones (**5**–**9**) were isolated from the aerial parts of *Mikania micrantha*. Their structures were determined by extensive spectroscopic analysis. Compound **4** features an adenine moiety in the molecule, which is the first nitrogen-containing sesquiterpenoid so far isolated from this plant species. Bioassays indicated that compounds **4** and **7**–**9** were active to show strong in vitro antibacterial activity toward all the seven tested bacteria with the MIC values ranging from 1.56 to 12.5 µg/mL. Notably, compounds **4** and **9** showed significant antibacterial activity against the drug-resistant bacterium of *MRSA* with MIC value close to reference compound vancomycin. Compounds **4** and **7**–**9** were further revealed to show in vitro cytotoxic activity toward human tumor A549, HepG2, MCF-7 and HeLa cell lines, with IC_50_ values ranging from 8.97 to 27.39 µM. No antibacterial and cytotoxic activity were displayed for the other compounds. The current research provided new data to support that *M. micrantha* is rich in structurally diverse bioactive compounds worthy of further development in medicinal or healthcare application and for crop-protection in agricultural fields.

## Figures and Tables

**Table 1:** ^1^H NMR data of compounds **1**–**4**, *δ* in ppm and *J* in Hz.

No	1 *^a^*	2 *^a^*	3 *^b^*	4 *^c^*
1	2.85, d (11.7)	3.07, dd (11.5, 2.0)	5.43, d (9.3)	2.77, br.d (11.1)
2	3.73, ddd (11.7, 9.7, 4.2)	α 1.75, ddd (14.2, 11.5, 4.0)	*α* 3.13, ddd (14.5, 9.3, 3.3)	*α* 1.22, m
		*β* 2.17, ddd (14.2, 2.2, 2.0)	*β* 2.57, dd (14.5, 3.1)	β 1.96, m
3	*α* 2.96, dd (12.6, 4.2)	4.88, dd (4.0, 2.2)	5.26, dd (3.3, 3.1)	*α* 2.42, dd (12.9, 5.3)
	*β* 2.58, dd (12.6, 9.7)			*β* 2.52, ddd (12.9, 10.2, 4.1)
5	7.79, br s	7.81, t (1.6)	8.12, d (1.2)	7.70, br.s
6	5.27, br s	5.33, br s	5.47, d (1.2)	4.93, br s
7	2.62, (13.9, 11.1)	2.61, dd (13.9, 10.0)	2.62, overlapped	2.81, m
8	4.64, ddd (11.1, 10.8, 4.6)	4.57, ddd (10.7, 10.0, 4.6)	5.51, dd (10.6, 10.2)	4.60, overlapped
9	*α* 2.06, dd (14.2, 10.8)	*α* 2.05, dd (14.3, 10.7)	5.29, d (10.6)	*α* 1.93, overlapped
	*β* 2.12, dd (14.2, 4.6)	*β* 2.10, dd (14.3, 4.6)		*β* 1.81, dd (13.8, 4.3)
11	2.90, dq (13.9, 7.0)	2.93, dq (13.9, 7.0)	2.97, dq (13.2, 6.9)	3.68, dt (12.1, 6.0)
13	1.40, d (7.0)	1.40, d (7.0)	1.38, d (6.9)	4.64, overlapped
				4.50, dd (14.5, 6.0)
14	1.25, s	1.19, s	2.09, s	0.98, s
2′				8.14, s
8′				8.06, s
-NH_2_				7.23, s

*^a^* Measured in methanol-*d*_4_; *^b^* Measured in pyridine-*d*_5_; *^c^* Measured in DMSO-*d*_6_.

**Table 2:** ^13^C NMR data of compounds **1**–**4**, *δ* in ppm.

No	1 *^a^*	2 *^a^*	3 *^b^*	4 *^c^*
1	66.9, CH	60.2, CH	64.4, CH	60.9, CH
2	67.1, CH	32.8, CH_2_	41.2, CH_2_	22.3, CH_2_
3	32.2, CH_2_	65.8, CH	65.7, CH	21.2, CH
4	131.1, C	138.0, C	138.6, C	131.1, C
5	151.4, CH	147.8, CH	145.6, CH	149.4, CH
6	81.5, CH	81.8, CH	79.3, CH	79.9, CH
7	55.2, CH	55.3, CH	51.8, CH	49.1, CH
8	79.2, CH	79.5, CH	75.6, CH	77.5, CH
9	44.4, CH_2_	43.8, CH_2_	121.8, CH	42.7, CH_2_
10	58.8, C	58.5, C	148.2, C	56.6, C
11	41.4, CH	41.2, CH	38.7, CH	45.0, CH
12	178.0, C	178.1, C	177.5, C	173.8, C
13	13.5, CH_3_	13.5, CH_3_	13.2, CH_3_	41.8, CH_2_
14	21.5, CH_3_	20.6, CH_3_	18.1, CH_3_	19.7, CH_3_
15	174.3, C	172.6, C	171.8, C	171.9, C
2′				152.6, CH
4′				149.7, C
5′				118.6, C
6′				156.0, C
8′				141.1, CH

*^a^* Measured in methanol-*d*_4_; *^b^* Measured in pyridine-*d*_5_; *^c^* Measured in DMSO-*d*_6_.

**Table 3:** Antibacterial activity of compounds **1**–**9** (MIC, µg/mL).

Compounds	*SA*	*MRSA*	*BC*	*CF*	*EC*	*ST*	*PS*
**1**	>100	>100	>100	>100	>100	>100	>100
**2**	>100	>100	>100	>100	>100	>100	>100
**3**	>100	>100	>100	>100	>100	>100	>100
**4**	6.25	6.25	3.125	1.56	6.25	6.25	6.25
**5**	>100	>100	>100	>100	>100	>100	>100
**6**	>100	>100	>100	>100	>100	>100	>100
**7**	6.25	12.5	6.25	3.125	6.25	6.25	3.125
**8**	6.25	12.5	6.25	3.125	6.25	6.25	3.125
**9**	6.25	6.25	3.125	3.125	6.25	3.125	3.125
**K**	1.56	50	0.78	1.56	1.56	0.78	1.56
**V**	1.56	3.125	1.56	3.125	1.56	0.78	1.56

K = Kanamycin sulfate; V = Vancomycin.

**Table 4:** Cytotoxic activity of compounds **1**–**9**, (IC_50_, µM) *^a^*.

No	A549	HepG2	MCF-7	HeLa
**1**	>100	>100	>100	>100
**2**	>100	>100	>100	>100
**3**	>100	>100	>100	>100
**4**	17.97 ± 0.89	15.62 ± 1.02	25.81 ± 1.22	27.39 ± 2.46
**5**	>100	>100	>100	>100
**6**	>100	>100	>100	>100
**7**	>100	>100	>100	>100
**8**	14.28 ± 0.71	16.36 ± 1.66	9.85 ± 0.64	13.92 ± 0.97
**9**	16.48 ± 1.35	18.06 ± 1.13	10.24 ± 0.95	11.79 ± 1.05
**10**	12.26 ± 1.32	15.84 ± 1.16	8.97 ± 0.93	10.75 ± 0.81
Adriamycin	0.68 ± 0.07	1.12 ± 0.16	0.86 ± 0.09	0.92 ± 0.11

*^a^* Values represent mean ± SD (n = 3) based on three individual experiments.

## Data Availability

Not applicable.

## Supplementary Materials

The following supporting information can be downloaded at: https://www.mdpi.com/article/10.3390/molecules28052119/s1, HR-ESI-MS, ^1^H-NMR, ^13^C-NMR, HSQC, and HMBC spectra of new compounds **1**–**4**.

## Sample Availability

Samples of the compounds are not available from the authors.
