# Supplementary material for: Germacrane Sesquiterpene Dilactones from Mikania micrantha and Their Antibacterial and Cytotoxic Activity
Source: Molecules. 2023 Feb 24;28(5):2119. doi: 10.3390/molecules28052119 (PMC10004635; doi:10.3390/molecules28052119)

Supporting Information

In this 'Supporting Information' file for the manuscript entitled "**Germacrane sesquiterpene dilactones from *Mikania micrantha* and their antibacterial and cytotoxic activity**", HR-ESI-MS, 1D and 2D NMR spectra of new compounds **1**, **2**, **3** and **4** are available here as listed below.

Contents:

Page 2–8: HR-ESI-MS, ^{1}H and ^{13}C NMR, HSQC, HMBC and NOESY spectra of compound **1**.

Page 9–15: HR-ESI-MS, ^{1}H and ^{13}C NMR, HSQC, HMBC and NOESY spectra of compound **2**.

Page 16–22: HR-ESI-MS, ^{1}H and ^{13}C NMR, HSQC, HMBC and NOESY spectra of compound **3**.

Page 23–29: HR-ESI-MS, ^{1}H and ^{13}C NMR, HSQC, HMBC and NOESY spectra of compound **4**.

HR-ESI-MS(−) of compound **1**

Mass Spectrum SmartFormula Report

Analysis Info

Analysis Name	D:\Data\MS\data\202101\donglimei_E7-2-3-2_neg_33_01_1351.d		
Method	LC_Direct Infusion_neg_100-1000mz.m	Operator	SCSIO
Sample Name	donglimei_E7-2-3-2_neg	Instrument / Ser#	maXis 29
Comment			

Acquisition Parameter

Source Type	ESI	Ion Polarity	Negative	Set Nebulizer	0.4 Bar
Focus	Active	Set Capillary	3800 V	Set Dry Heater	180 °C
Scan Begin	100 m/z	Set End Plate Offset	-500 V	Set Dry Gas	4.0 l/min
Scan End	2000 m/z	Set Collision Cell RF	550.0 Vpp	Set Divert Valve	Waste

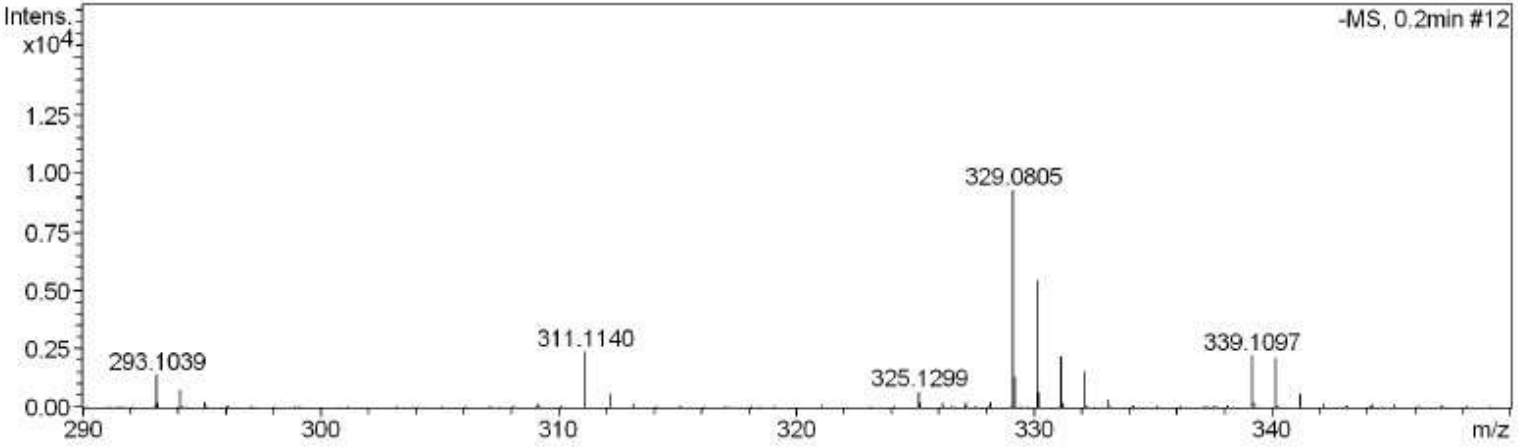

Meas. m/z	#	Formula	Score	m/z	err [ppm]	err [mDa]	mSigma	rdb	e⁻ Conf	N-Rule
293.1039	1	C 15 H 17 O 6	100.00	293.1031	-3.0	-0.9	207.7	7.5	even	ok
329.0805	1	C 15 H 18 Cl O 6	100.00	329.0797	-2.4	-0.8	202.6	6.5	even	ok

^{1}H-NMR spectrum of compound **1**

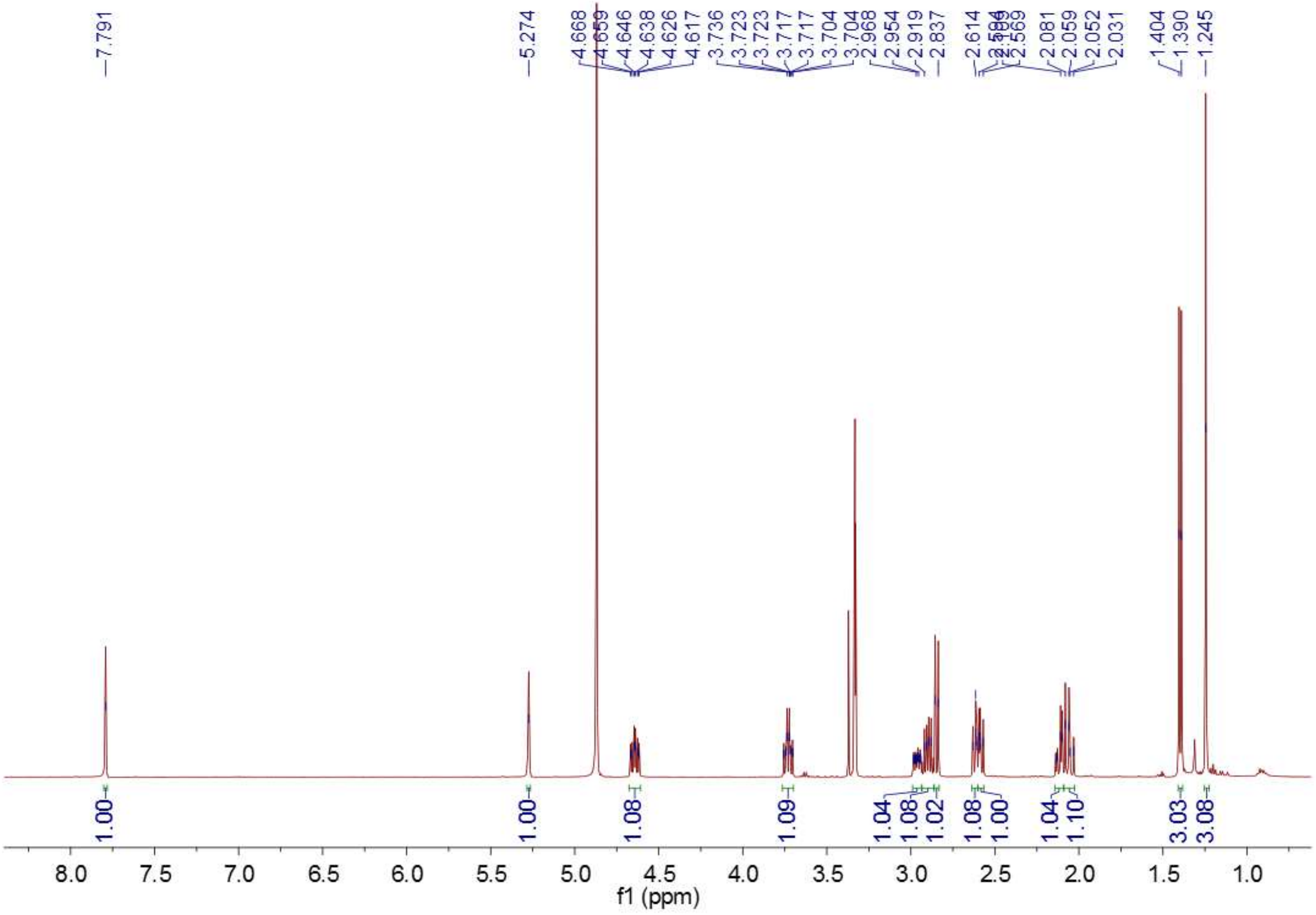

^{13}C-NMR spectrum of compound **1**

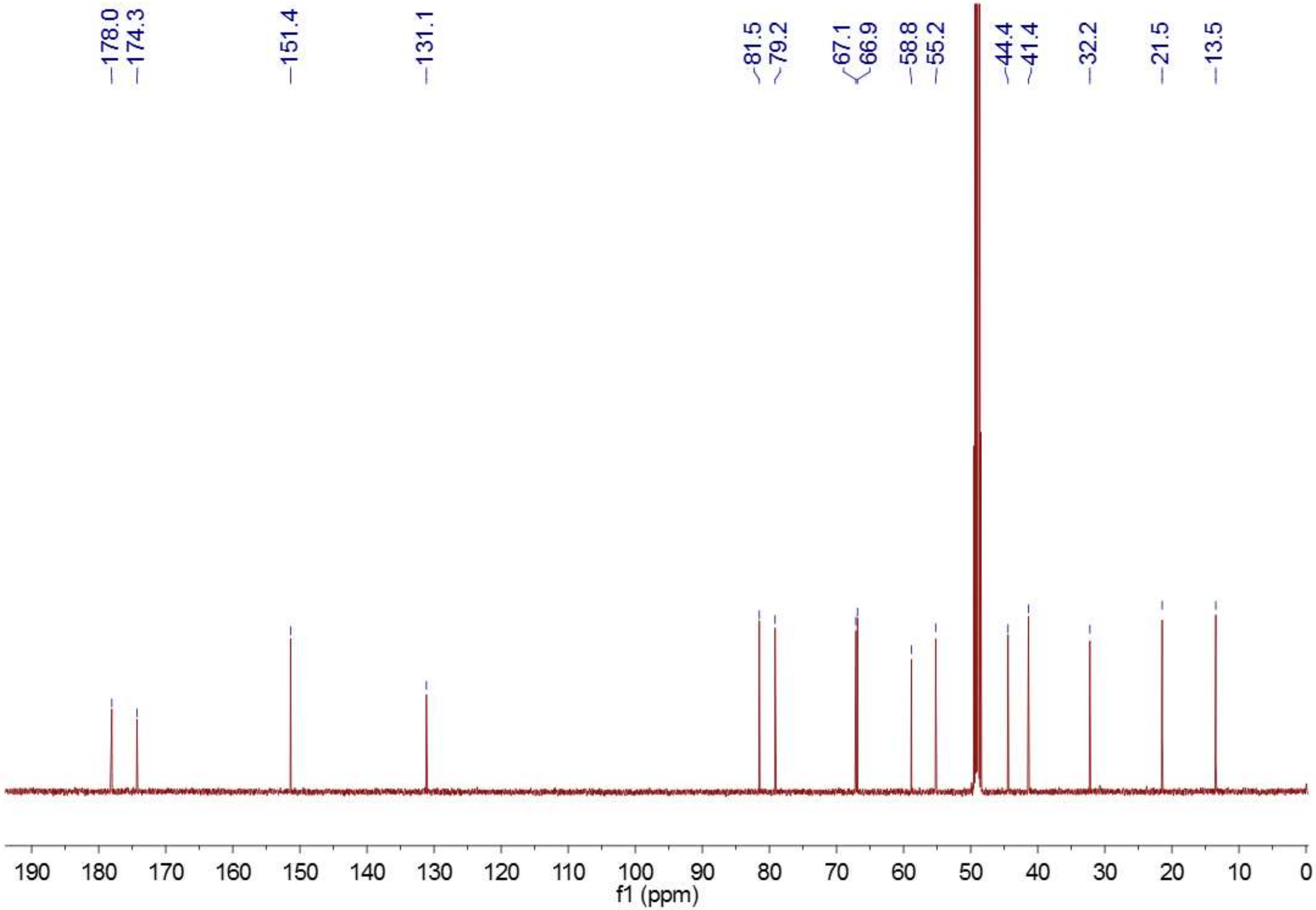

HSQC spectrum of compound **1**

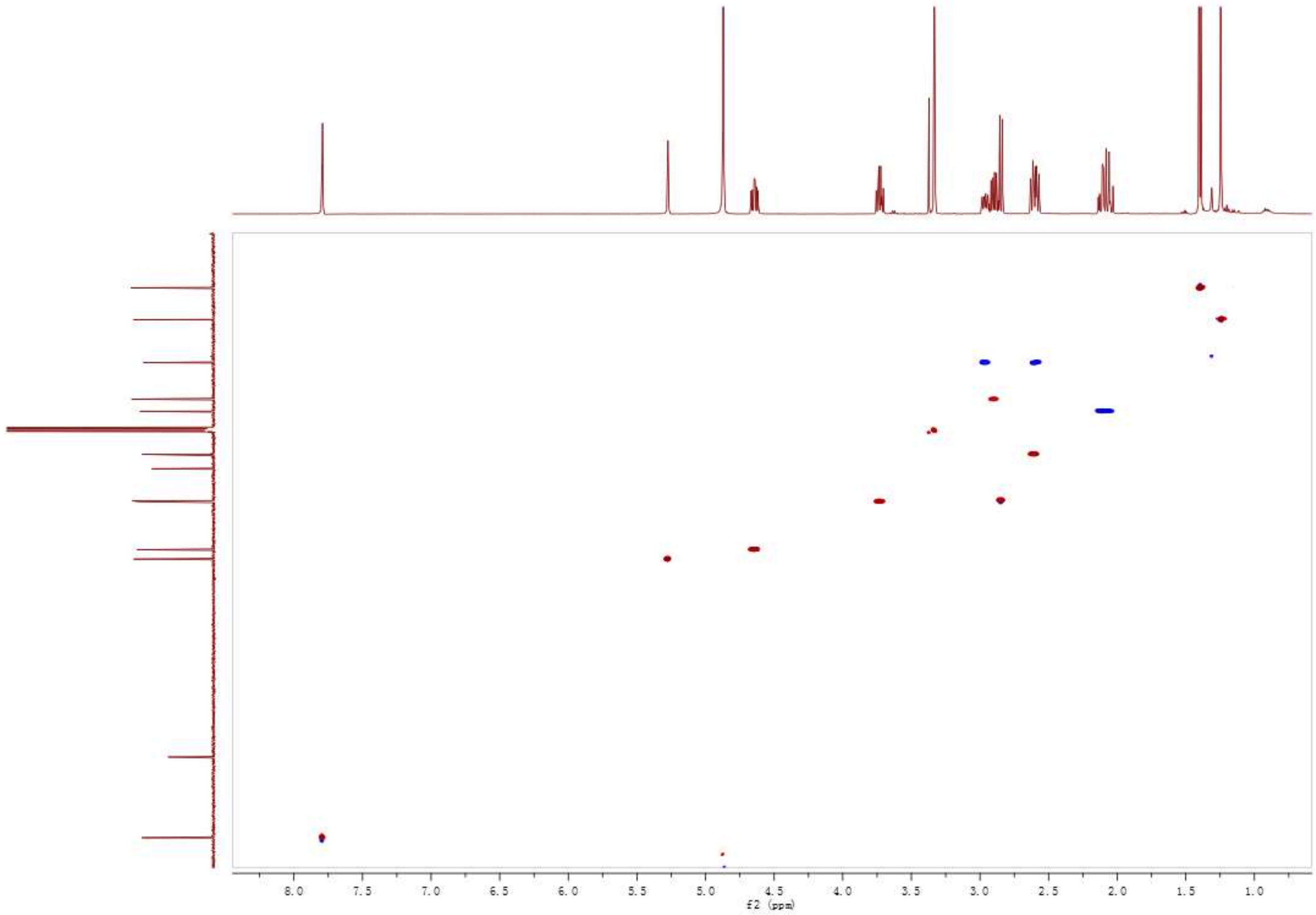

^{1}H-^{1}H COSY spectrum of compound **1**

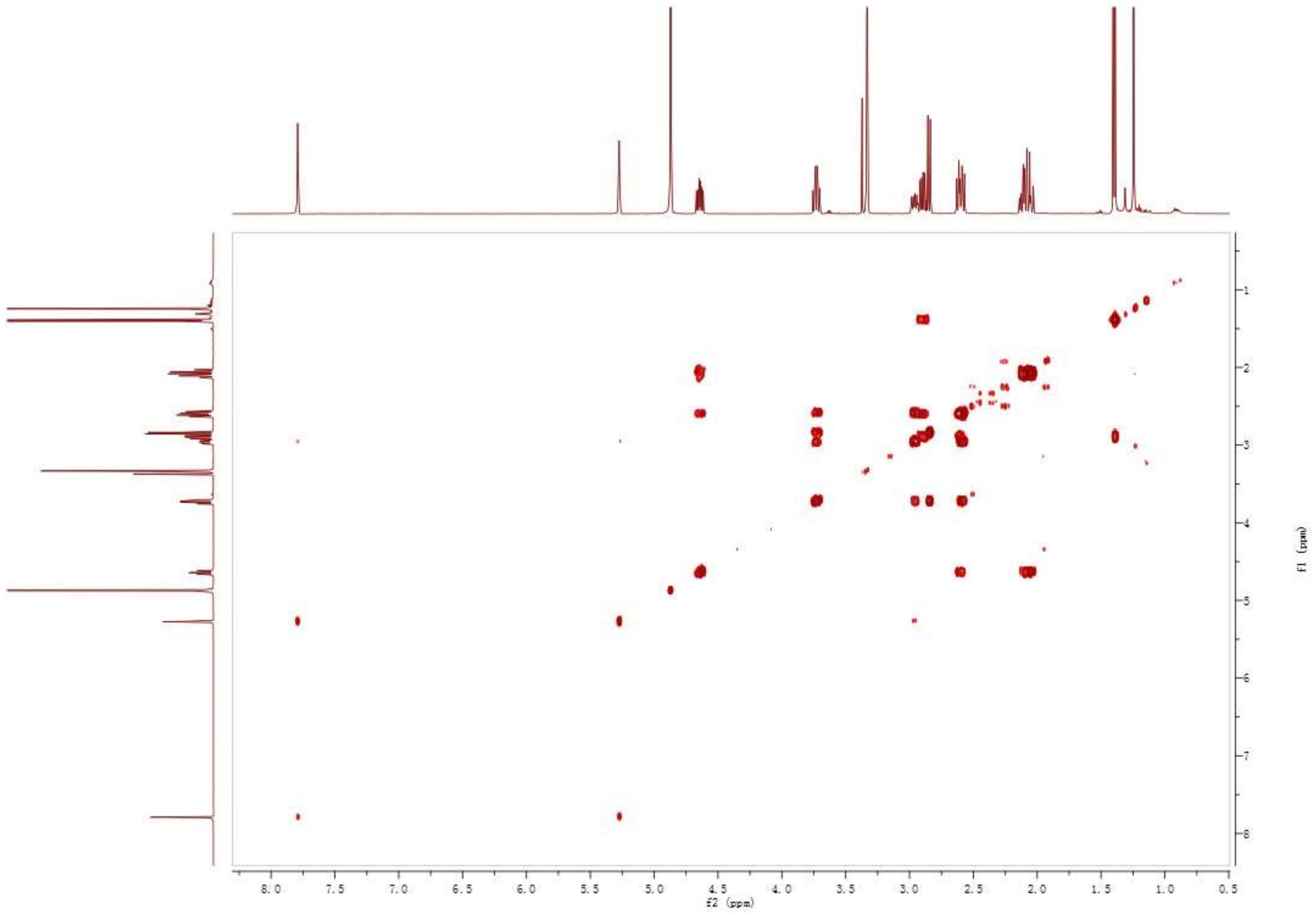

HMBC spectrum of compound **1**

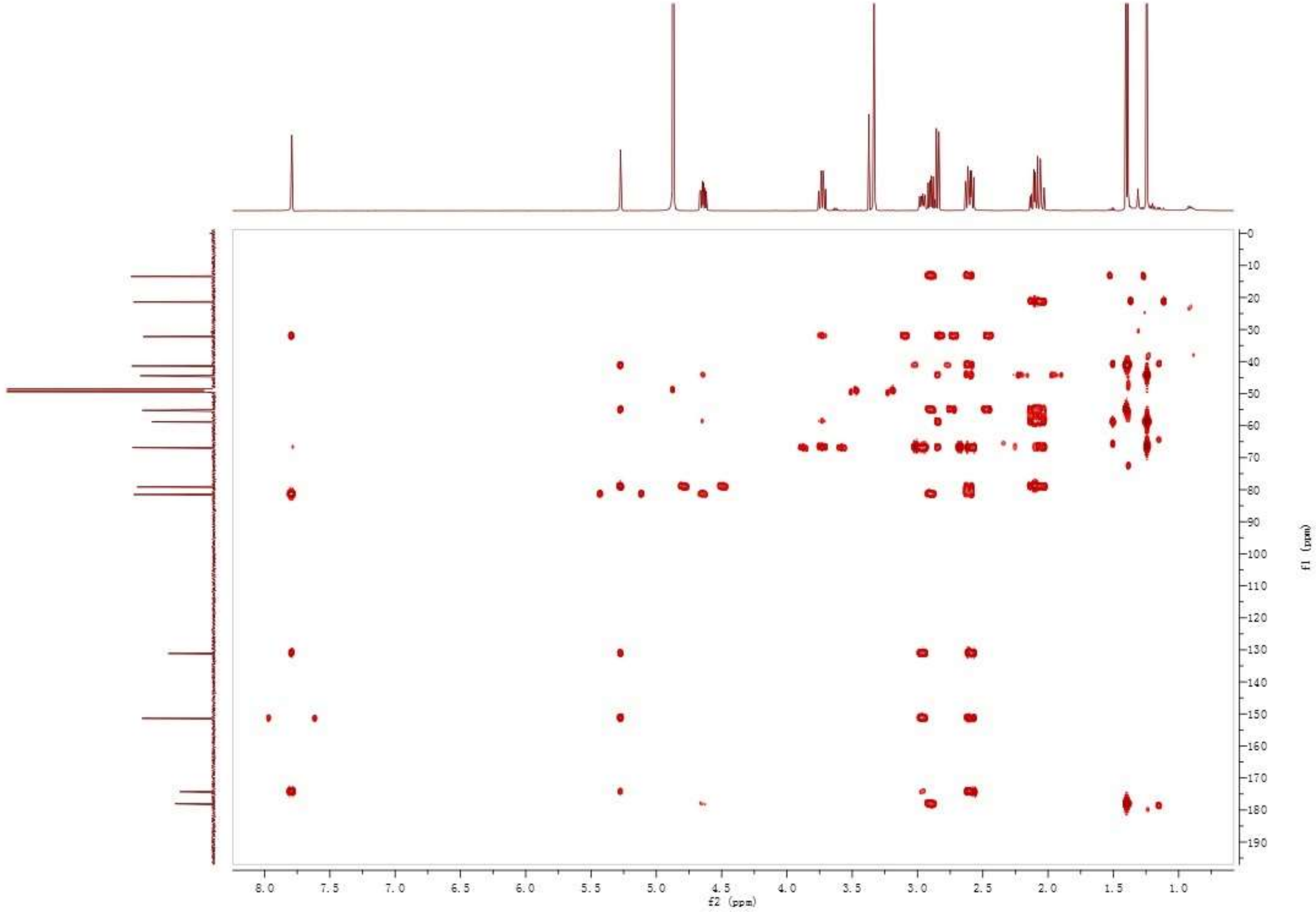

NOESY spectrum of compound **1**

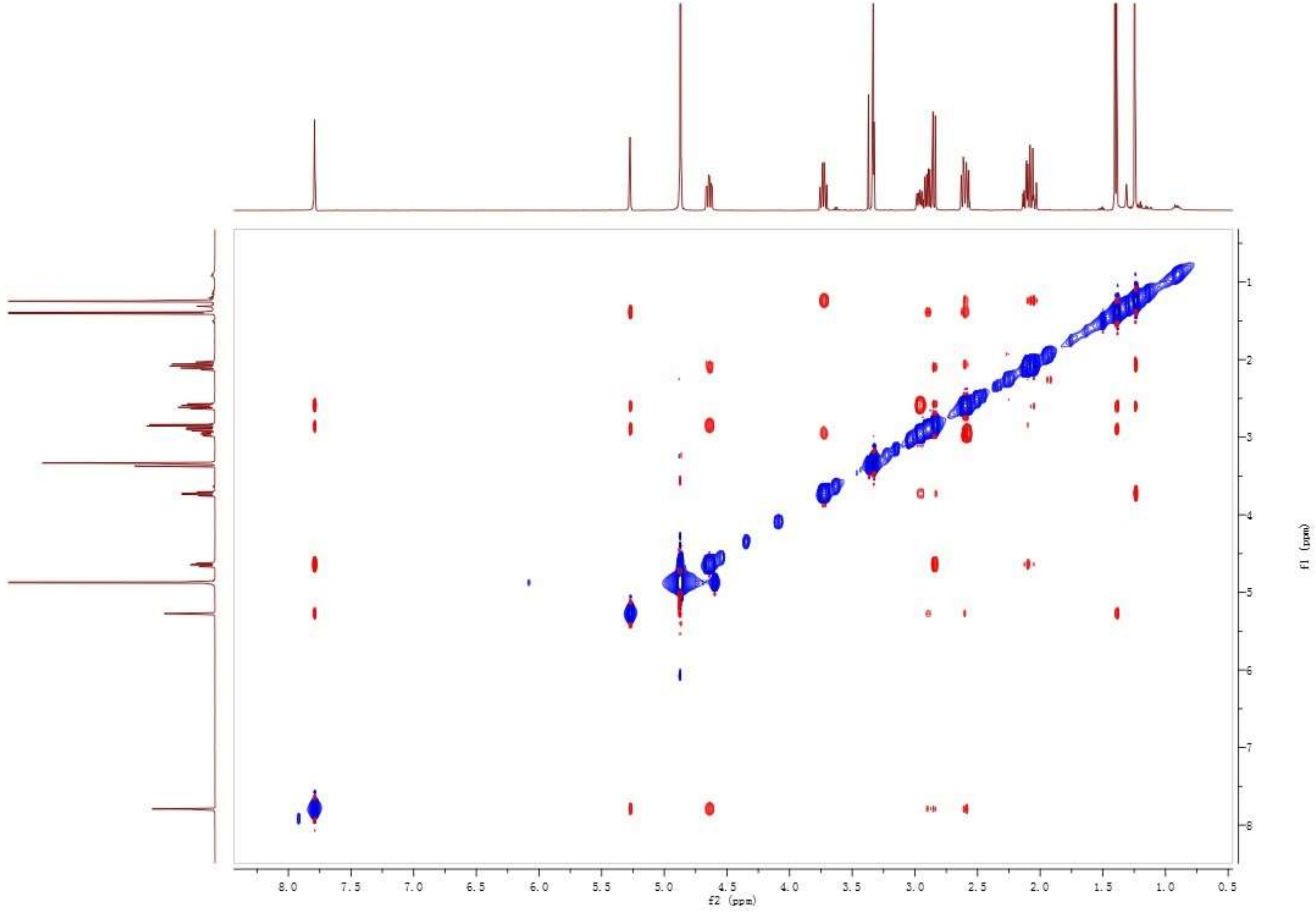

HR-ESI-MS(−) of compound **2**

Mass Spectrum SmartFormula Report

Analysis Info

Analysis Name	D:\Data\MS\data\202112\donglimei_MM-B-15_neg.d			
Method	tune_low_neg_20210914.m	Operator	SCSIO	
Sample Name	donglimei_mm-B-15_neg	Instrument	maXis	255552.00029
Comment				

Acquisition Parameter

Source Type	ESI	Ion Polarity	Negative	Set Nebulizer	0.4 Bar
Focus	Active	Set Capillary	4000 V	Set Dry Heater	180 °C
Scan Begin	100 m/z	Set End Plate Offset	-500 V	Set Dry Gas	4.0 l/min
Scan End	2000 m/z	Set Charging Voltage	0 V	Set Divert Valve	Waste
		Set Corona	0 nA	Set APCI Heater	0 °C

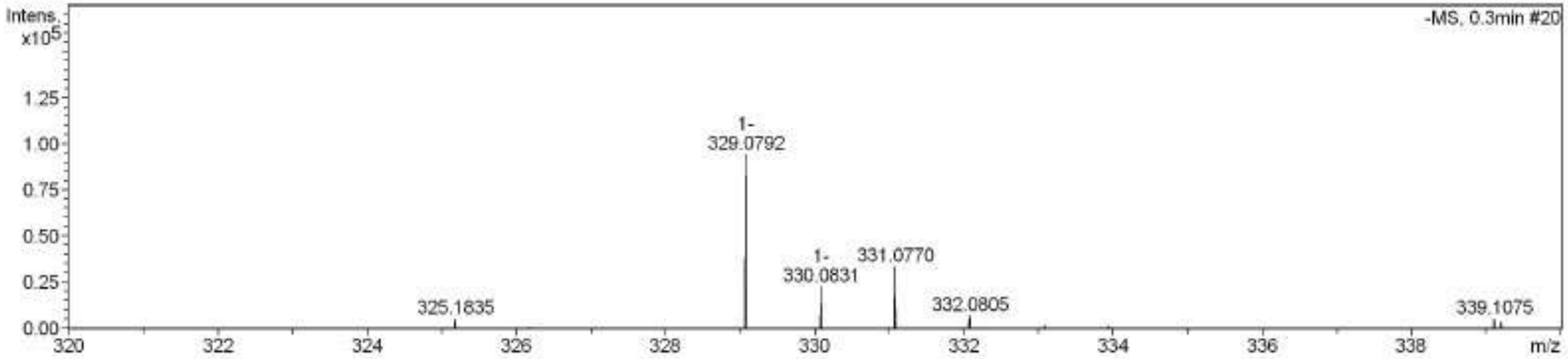

Meas. m/z	#	Ion Formula	Score	m/z	err [ppm]	err [mDa]	mSigma	rdb	e⁻ Conf	N-Rule
329.079221	1	C15H18ClO6	100.00	329.079740	1.6	0.5	35.1	6.5	even	ok
623.188965	1	C30H36ClO12	100.00	623.190078	-1.8	-1.1	57.5	12.5	even	ok

^{1}H-NMR spectrum of compound **2**

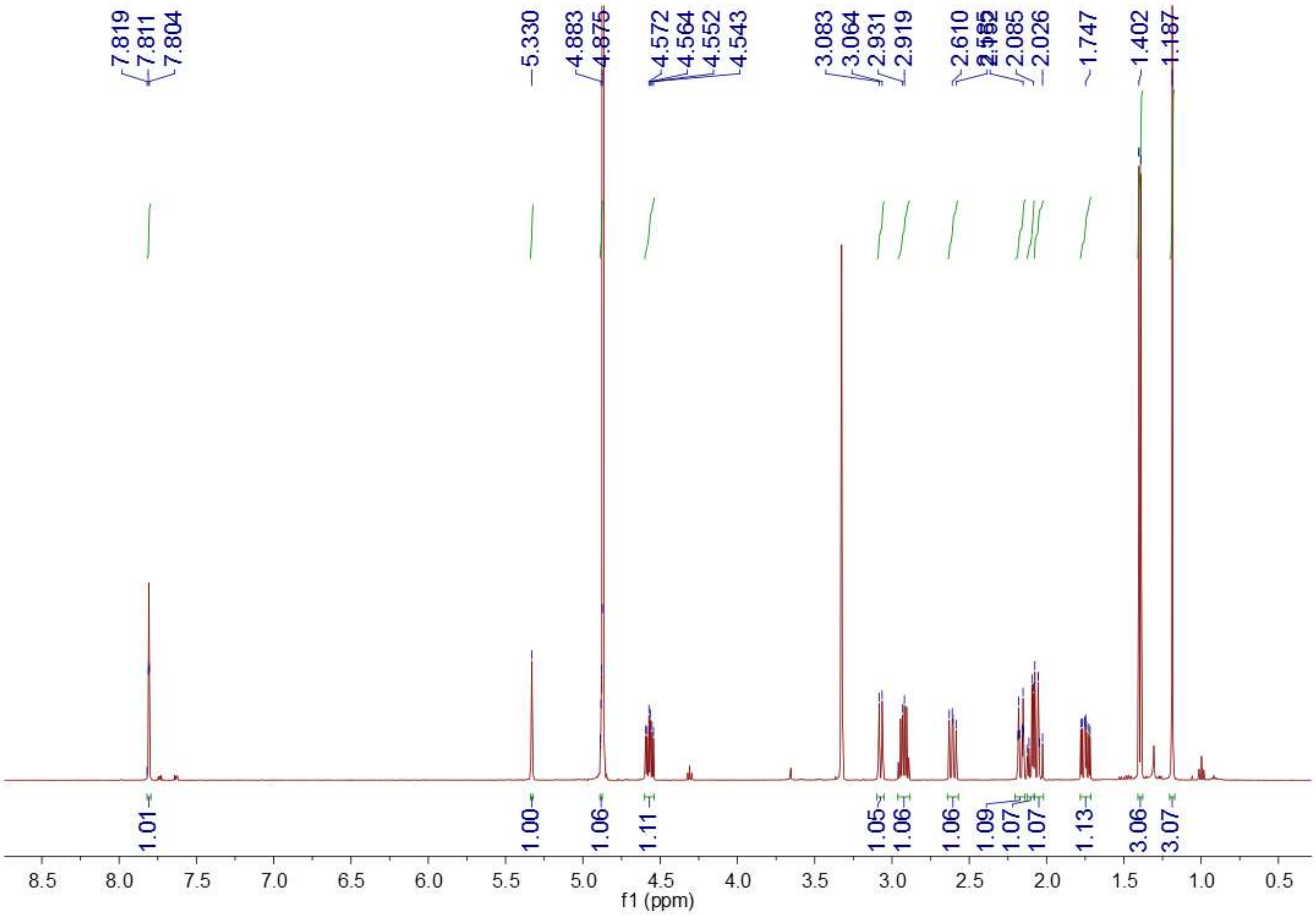

^{13}C-NMR spectrum of compound **2**

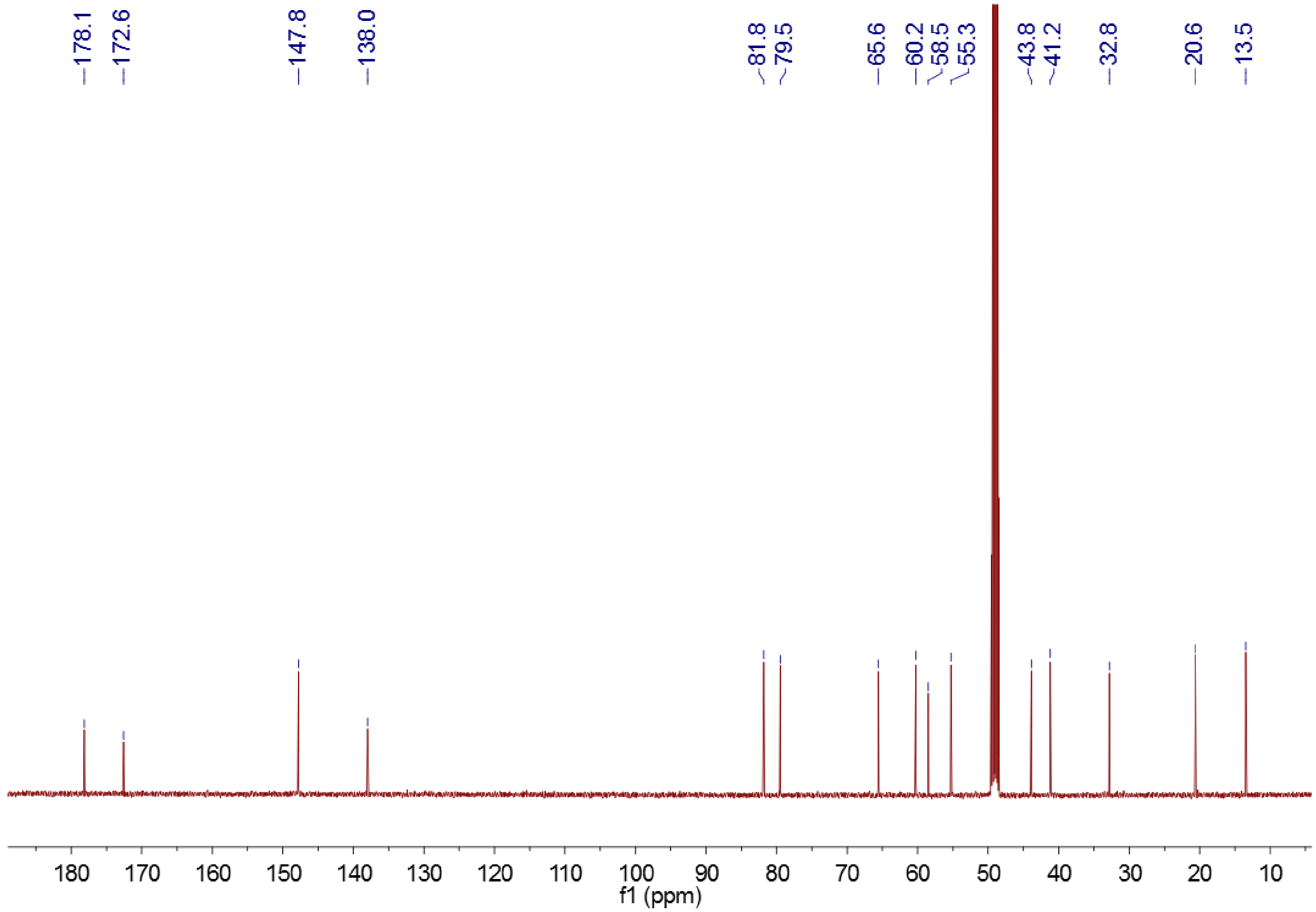

HSQC spectrum of compound **2**

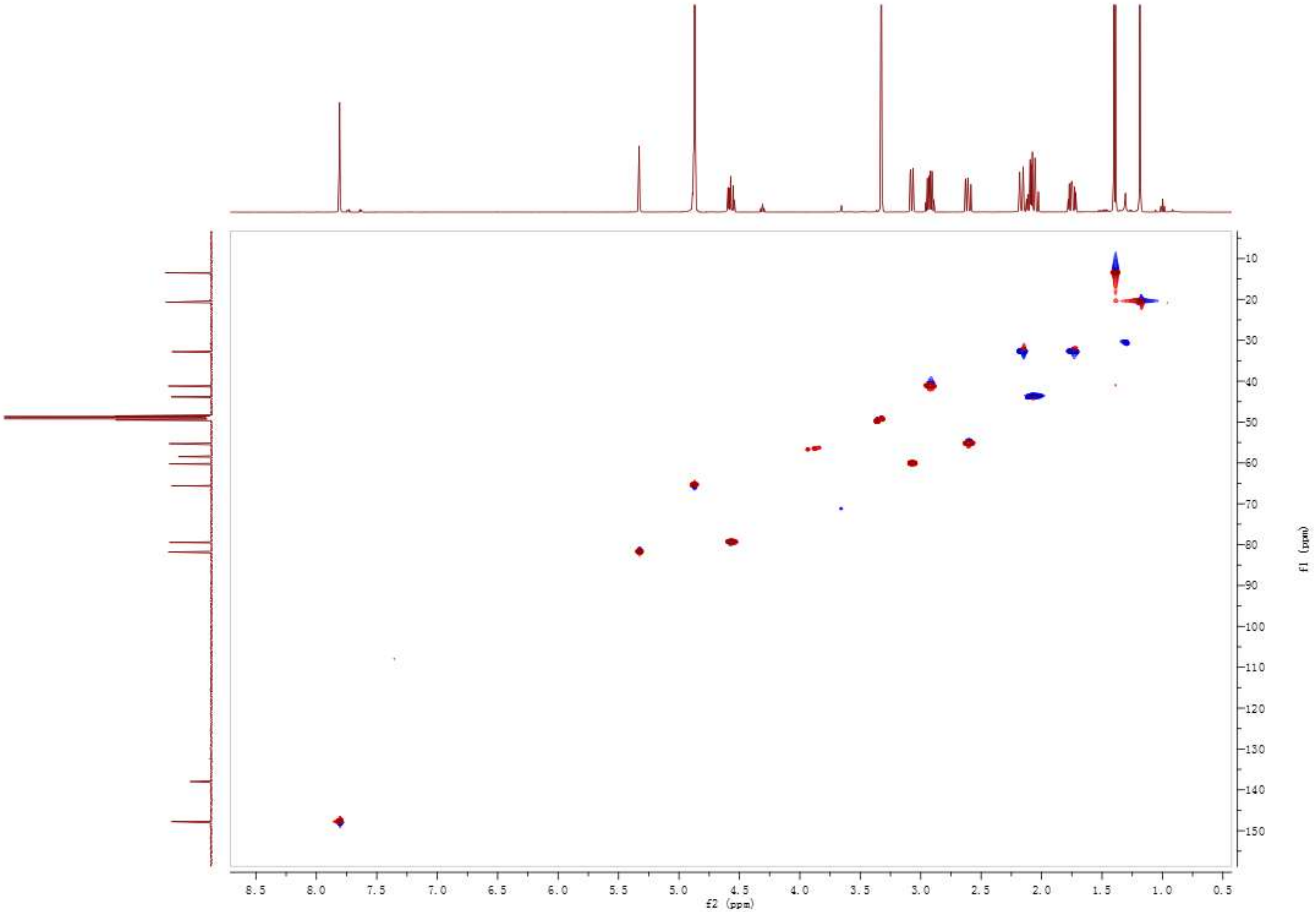

^{1}H-^{1}H COSY spectrum of compound **2**

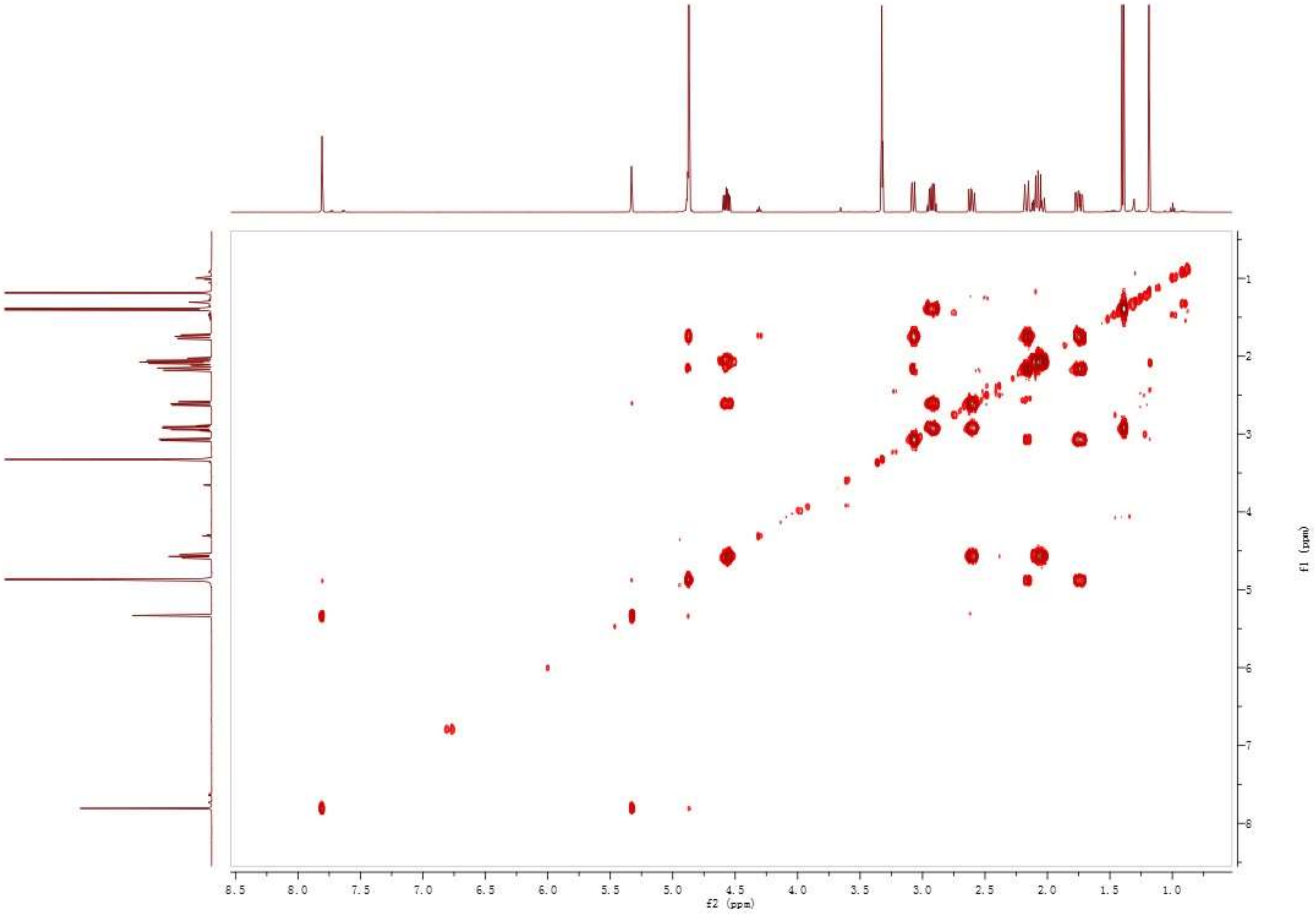

HMBC spectrum of compound **2**

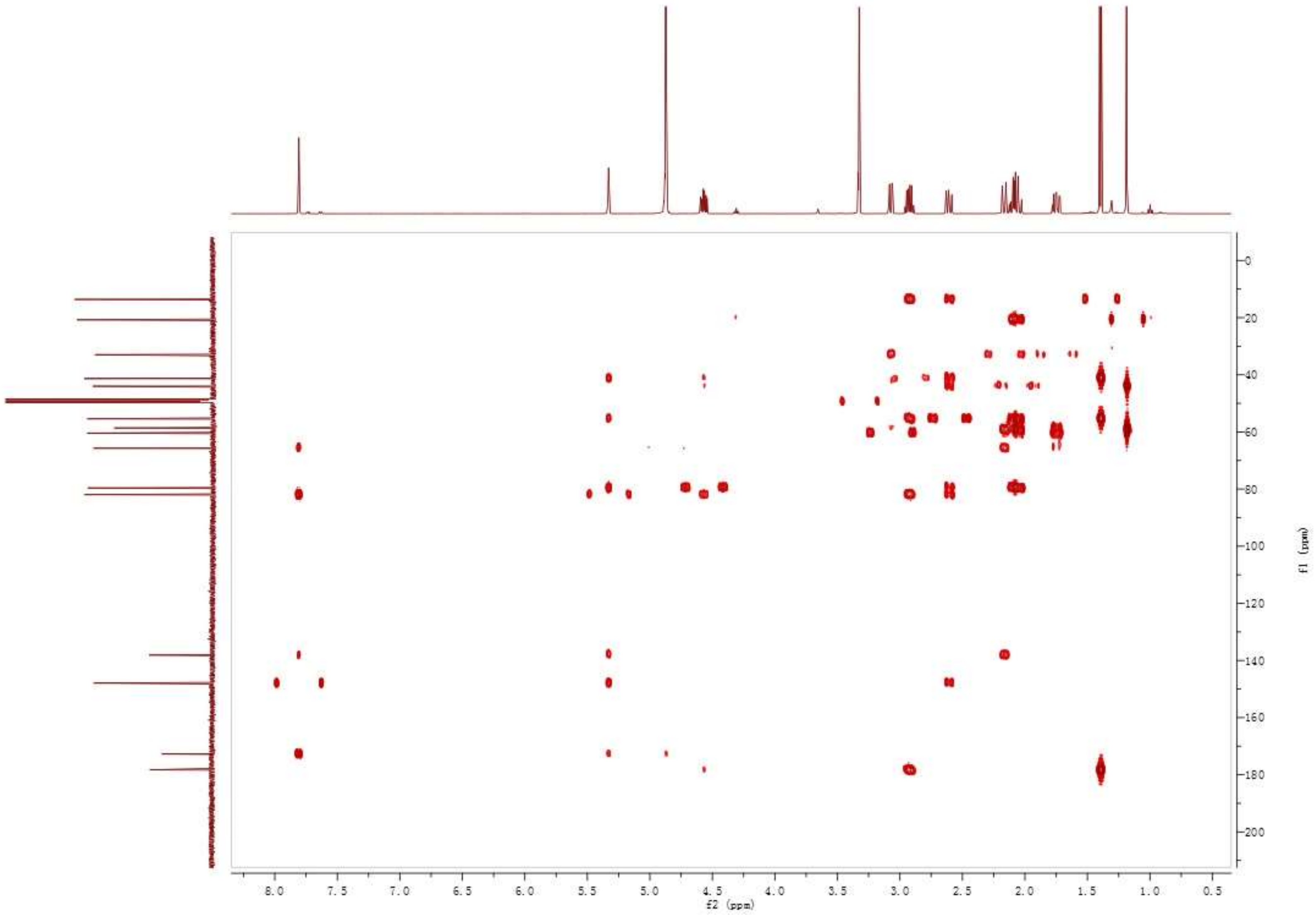

NOESY spectrum of compound **2**

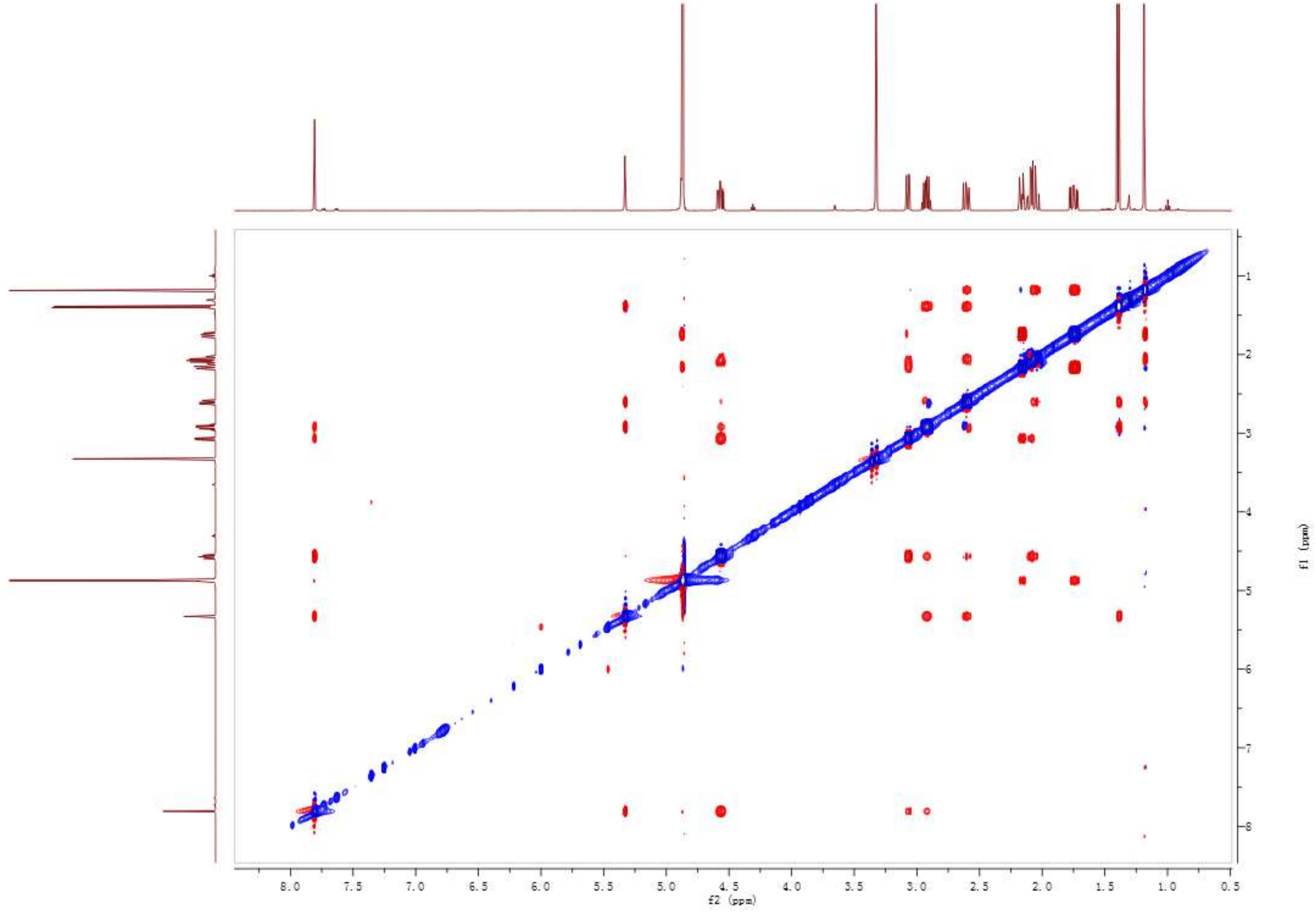

HR-ESI-MS(+) of compound **3**

Mass Spectrum SmartFormula Report

Analysis Info

Analysis Name	D:\Data\MS\data\202101\donglimei_E8-5-3-3_neg_24_01_1342.d		
Method	LC_Direct Infusion_neg_100-1000mz.m	Operator	SCSIO
Sample Name	donglimei_E8-5-3-3_neg	Instrument / Ser#	maXis 29
Comment			

Acquisition Parameter

Source Type	ESI	Ion Polarity	Negative	Set Nebulizer	0.4 Bar
Focus	Active	Set Capillary	3800 V	Set Dry Heater	180 °C
Scan Begin	100 m/z	Set End Plate Offset	-500 V	Set Dry Gas	4.0 l/min
Scan End	2000 m/z	Set Collision Cell RF	550.0 Vpp	Set Divert Valve	Waste

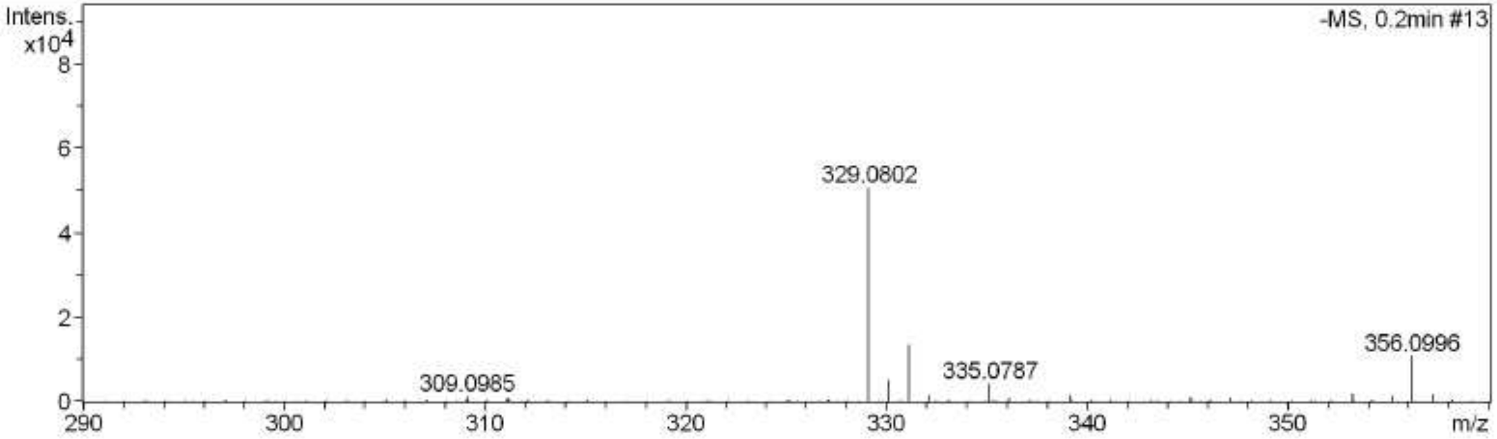

Meas. m/z	#	Formula	Score	m/z	err [ppm]	err [mDa]	mSigma	rdb	e¯ Conf	N-Rule
293.1036	1	C 15 H 17 O 6	100.00	293.1031	-1.8	-0.5	77.0	7.5	even	ok
329.0802	1	C 15 H 18 Cl O 6	100.00	329.0797	-1.5	-0.5	46.0	6.5	even	ok
623.1912	1	C 30 H 36 Cl O 12	100.00	623.1901	-1.9	-1.2	47.5	12.5	even	ok

^{1}H-NMR spectrum of compound **3**

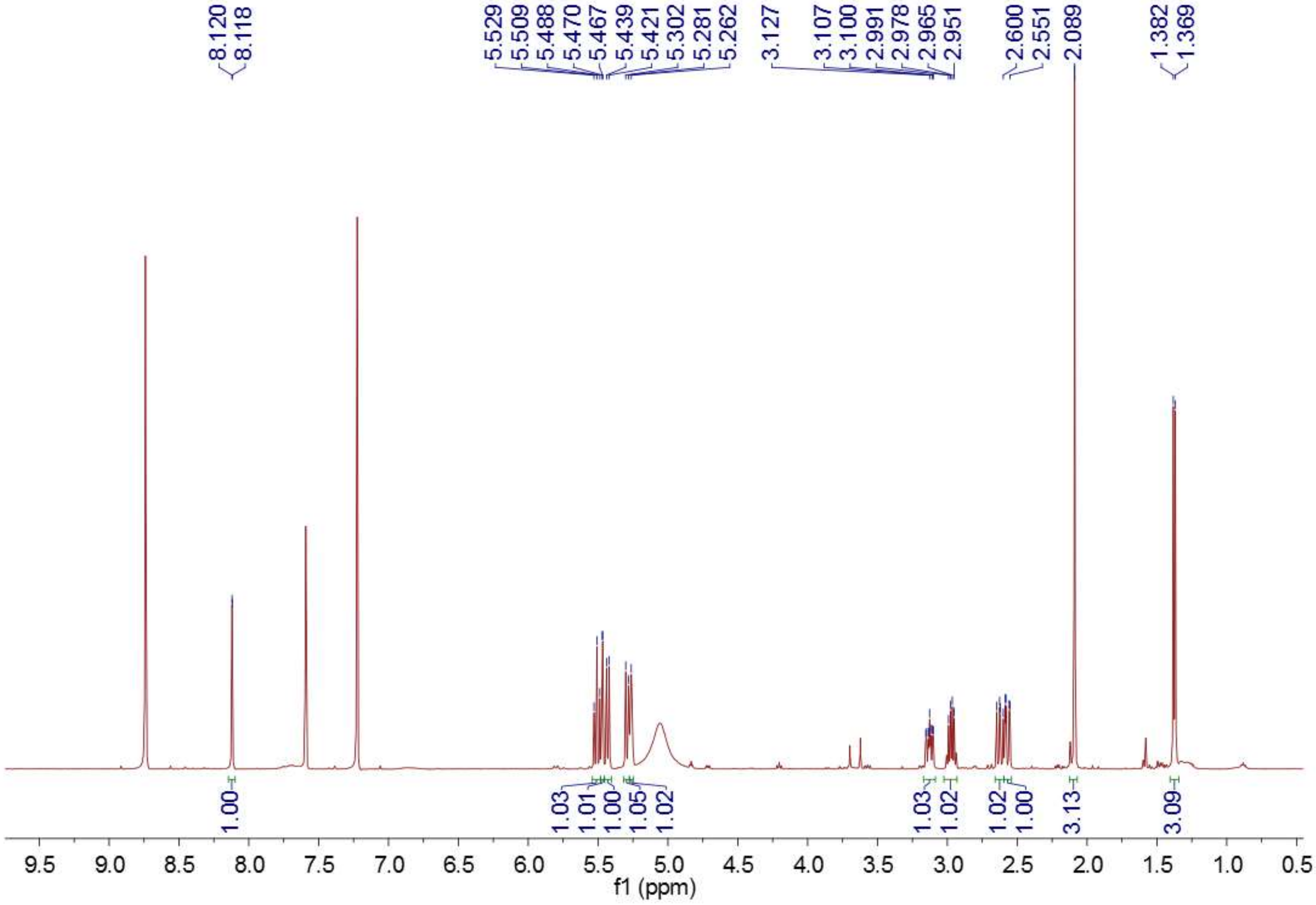

^{13}C-NMR spectrum of compound **3**

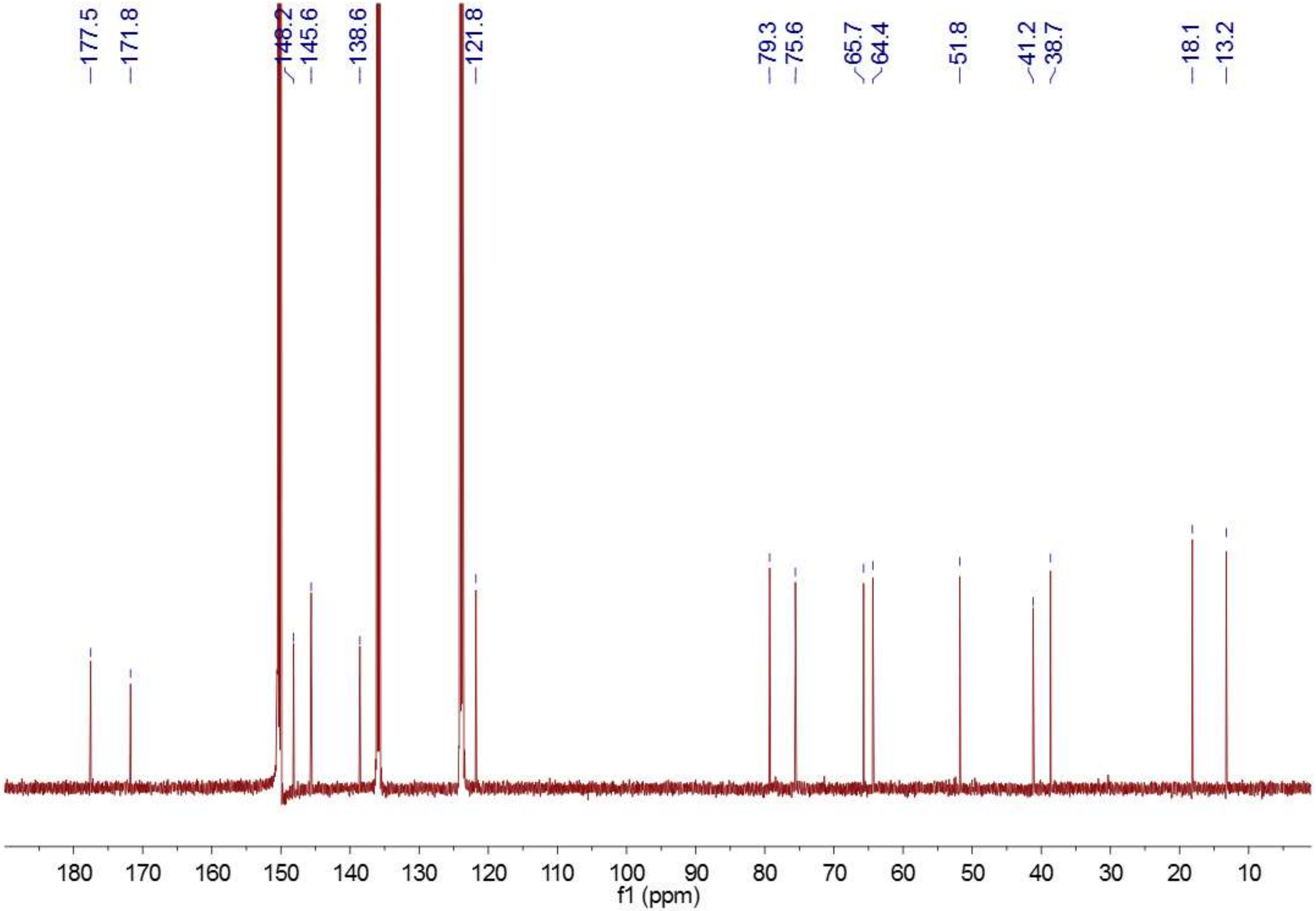

HSQC spectrum of compound **3**

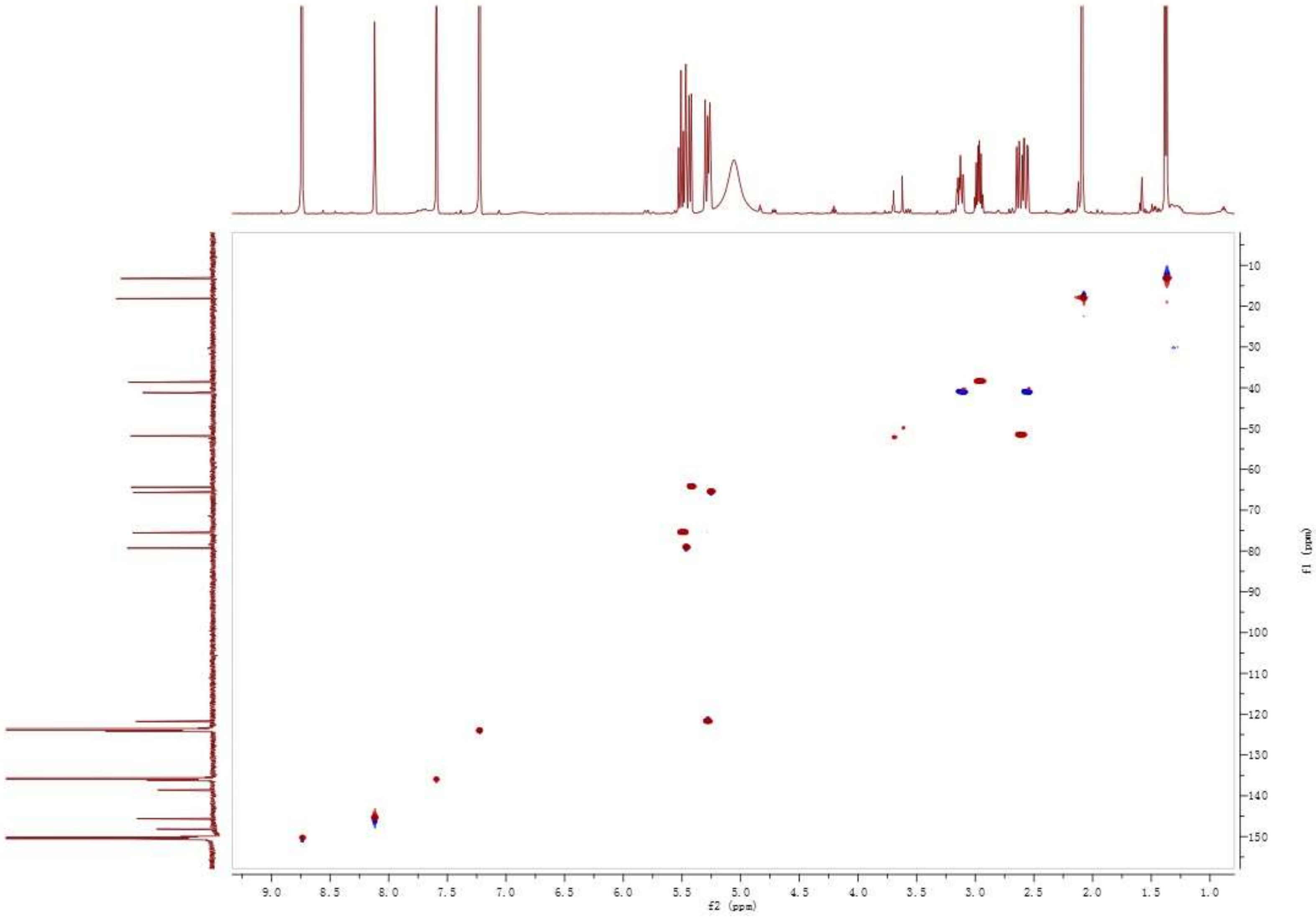

^{1}H-^{1}H COSY spectrum of compound **3**

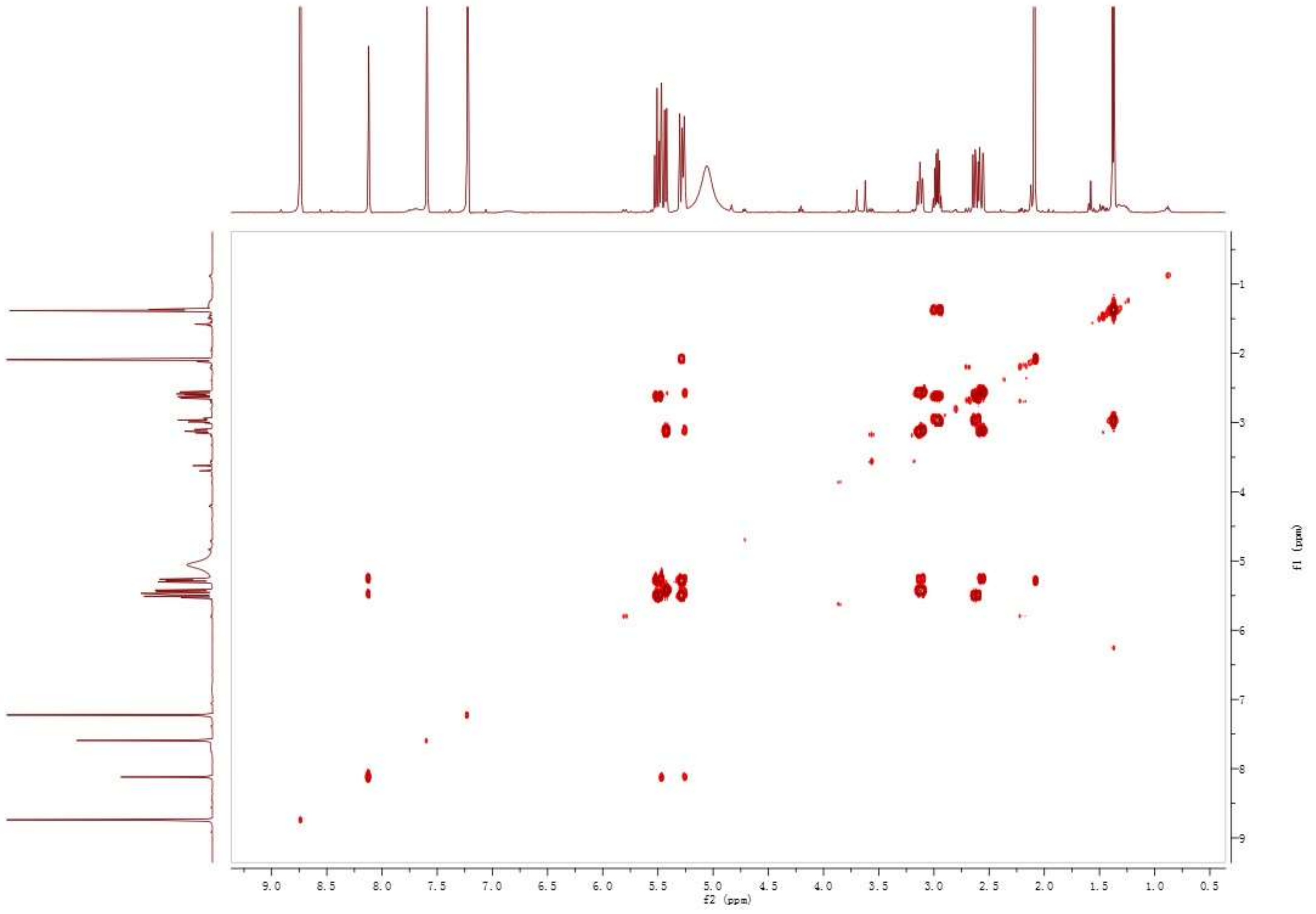

HMBC spectrum of compound **3**

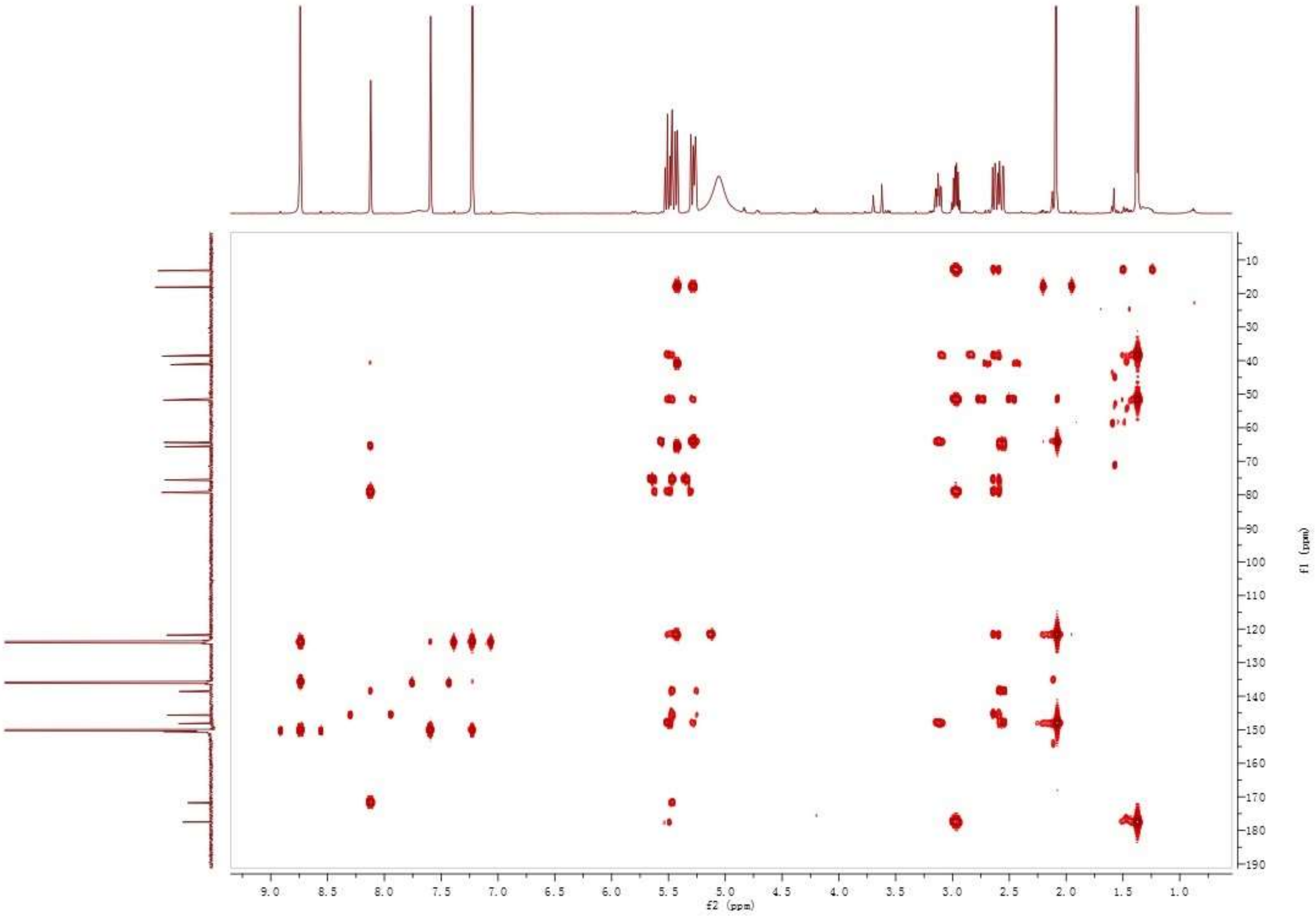

NOESY spectrum of compound **3**

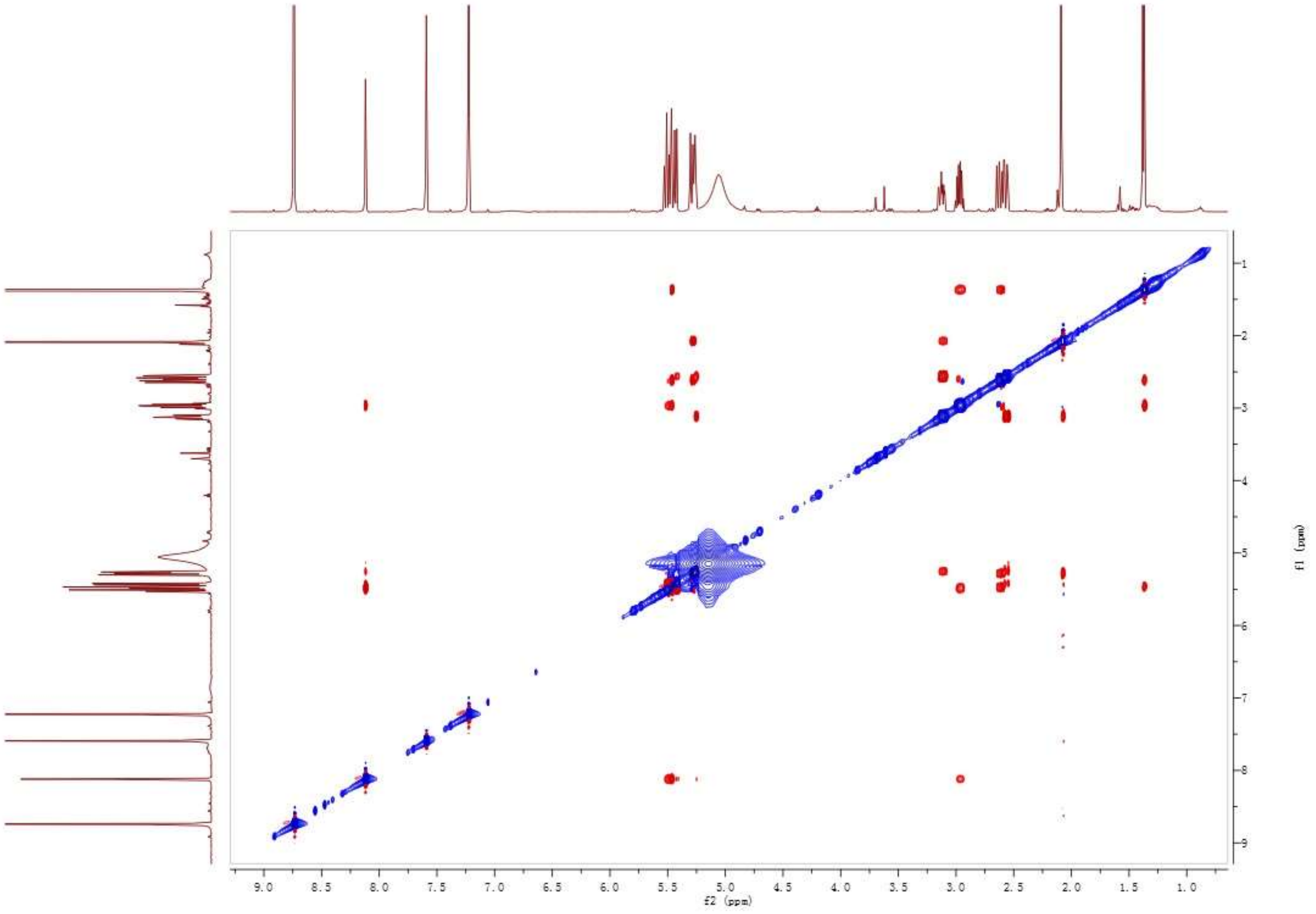

HR-ESI-MS(−) of compound **4**

Mass Spectrum SmartFormula Report

Analysis Info

Analysis Name	D:\Data\MS\data\202101\donglimei_E9-4-3_neg_34_01_1352.d		
Method	LC_Direct Infusion_neg_100-1000mz.m	Operator	SCSIO
Sample Name	donglimei_E9-4-3_neg	Instrument / Ser#	maXis 29
Comment			

Acquisition Parameter

Source Type	ESI	Ion Polarity	Negative	Set Nebulizer	0.4 Bar
Focus	Active	Set Capillary	3800 V	Set Dry Heater	180 °C
Scan Begin	100 m/z	Set End Plate Offset	-500 V	Set Dry Gas	4.0 l/min
Scan End	2000 m/z	Set Collision Cell RF	550.0 Vpp	Set Divert Valve	Waste

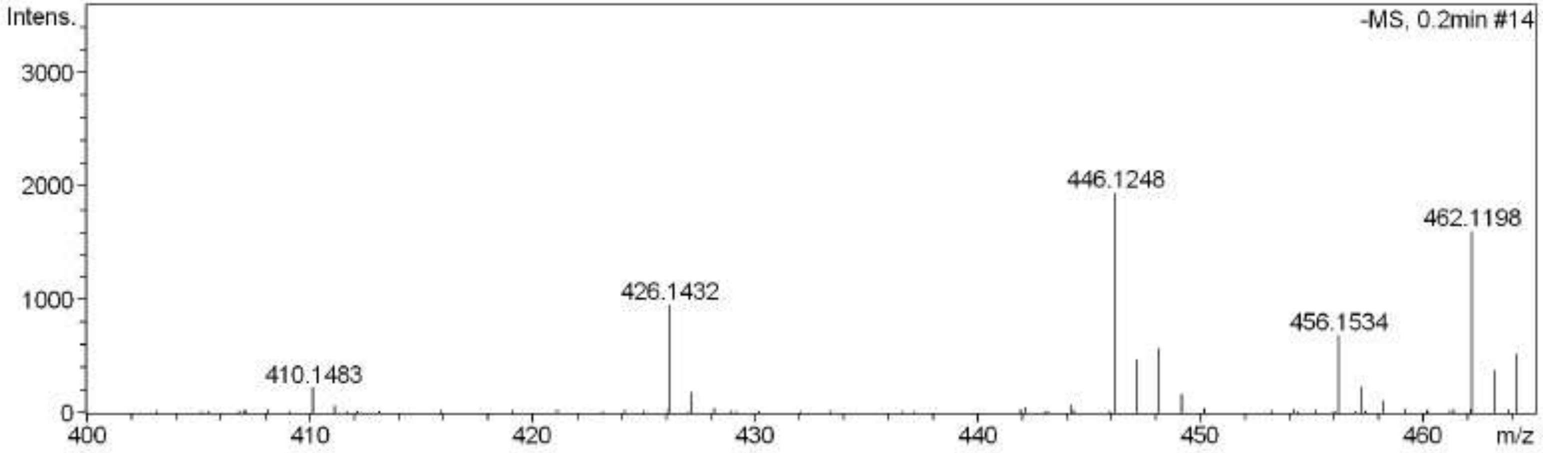

Meas. m/z	#	Formula	Score	m/z	err [ppm]	err [mDa]	mSigma	rdb	e⁻ Conf	N-Rule
410.1483	1	C 21 H 16 N 9 O	100.00	410.1483	0.0	0.0	12.9	18.5	even	ok
446.1248	1	C 20 H 21 Cl N 5 O 5	100.00	446.1237	-2.6	-1.2	27.3	12.5	even	ok

^{1}H-NMR spectrum of compound **4**

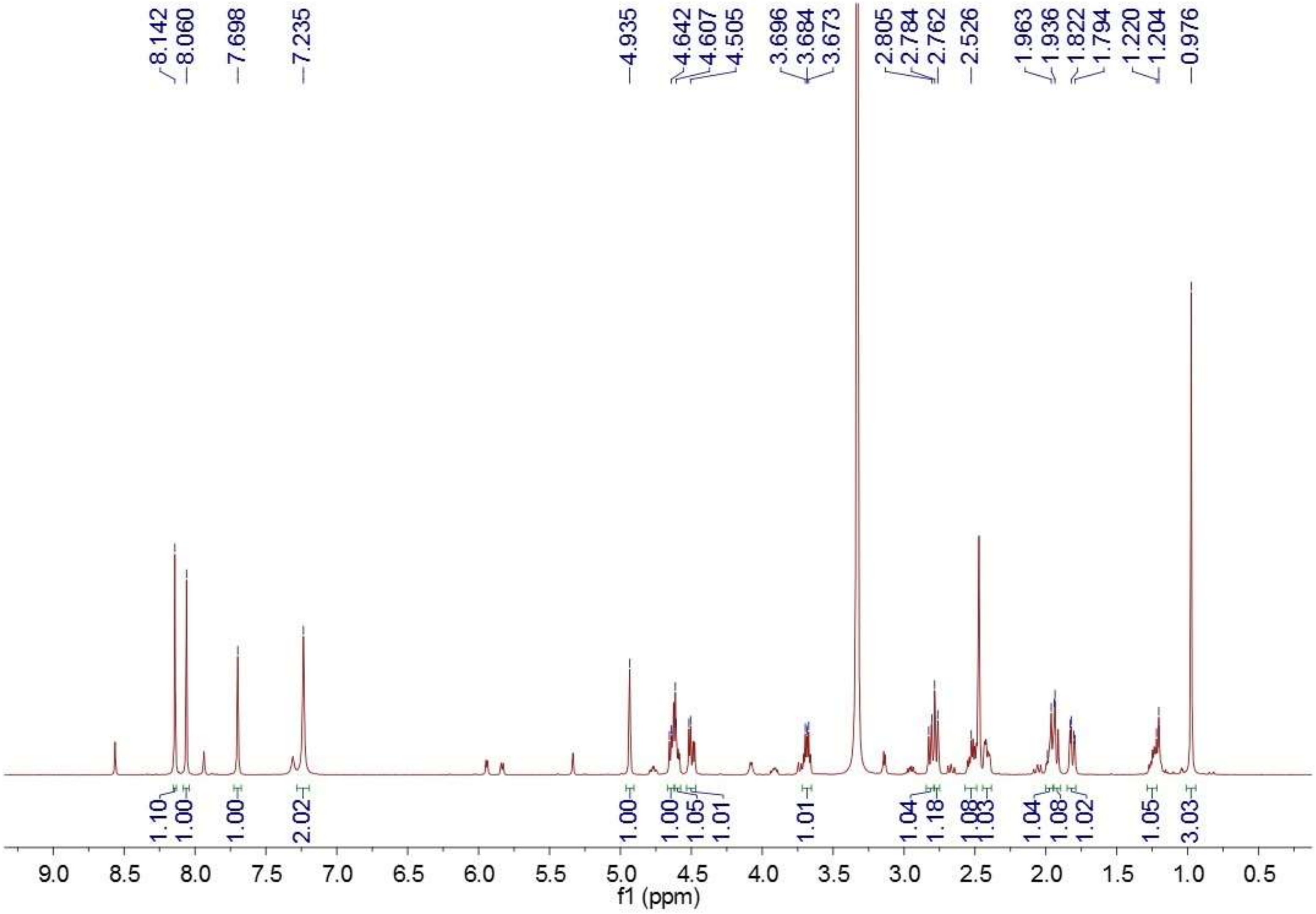

13C-NMR spectrum of compound **4**

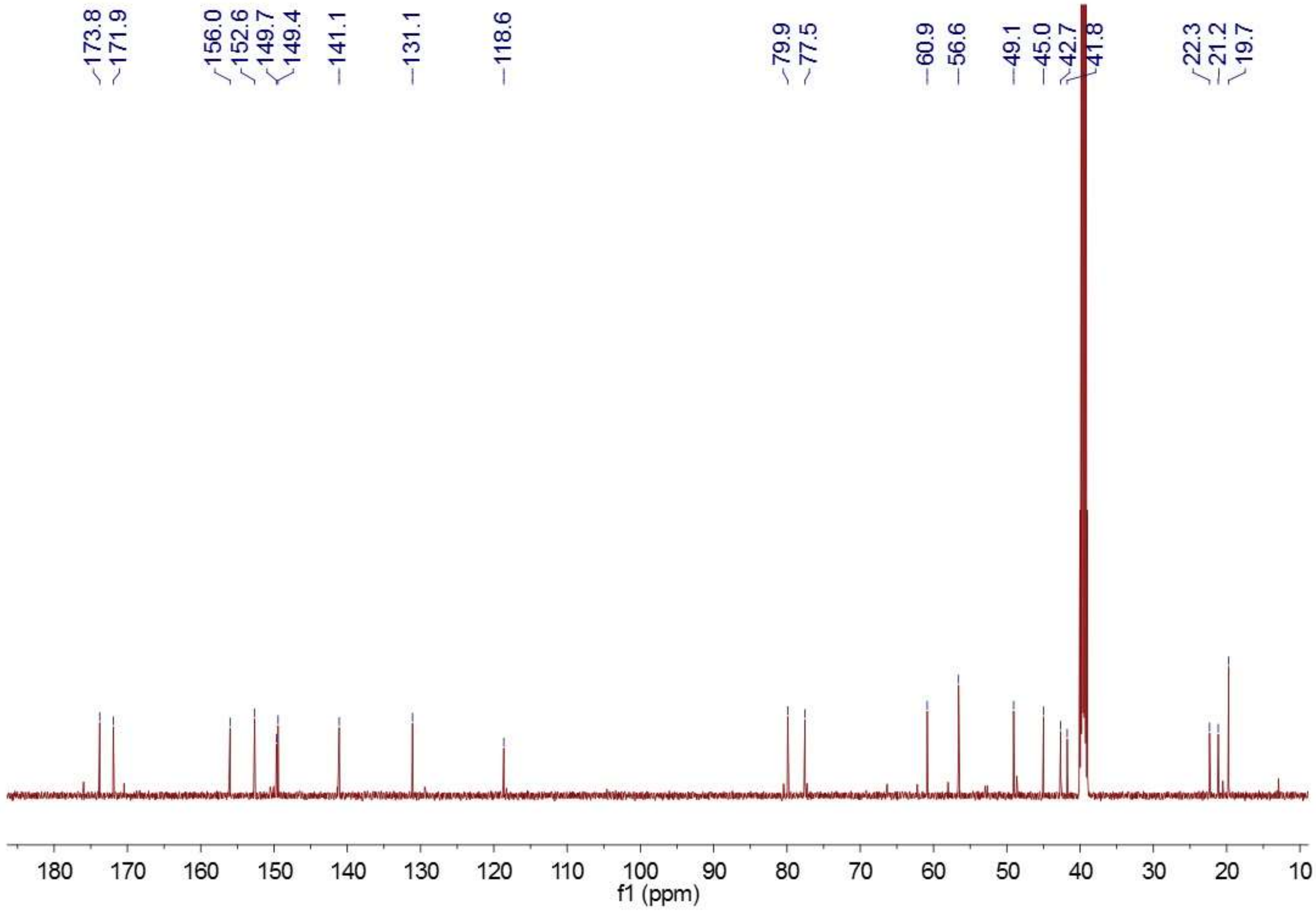

HSQC spectrum of compound **4**

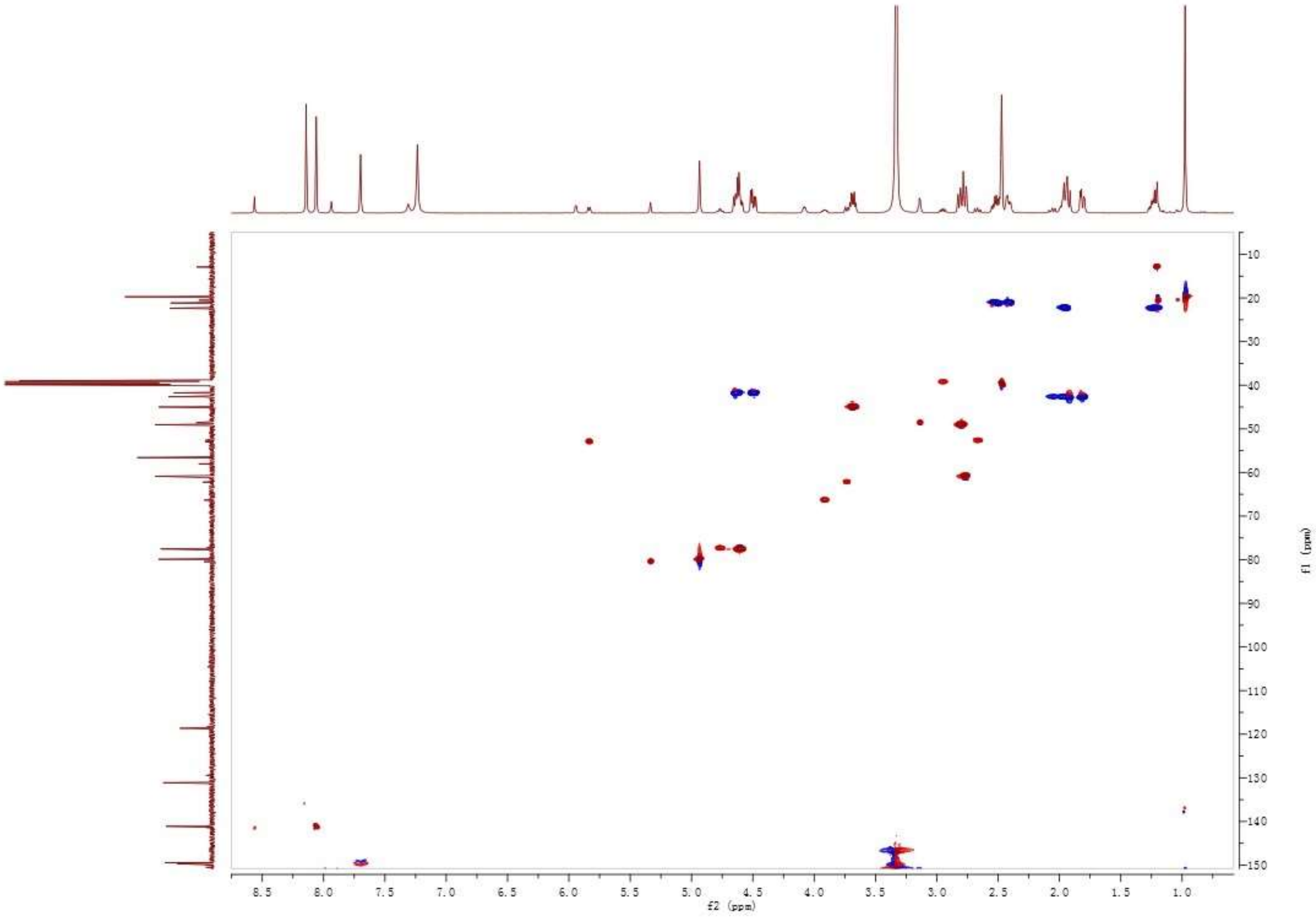

^{1}H-^{1}H COSY spectrum of compound **4**

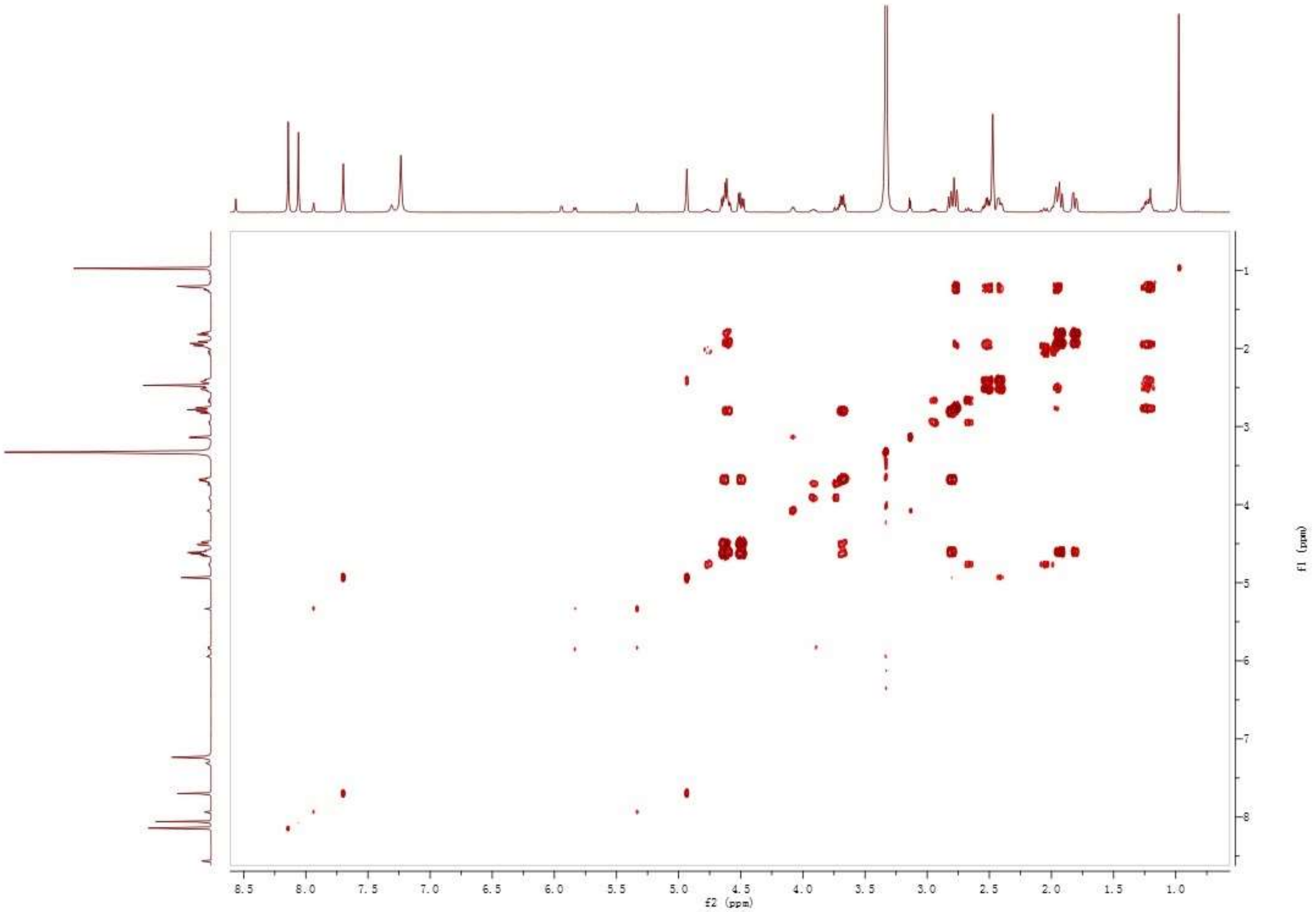

HMBC spectrum of compound **4**

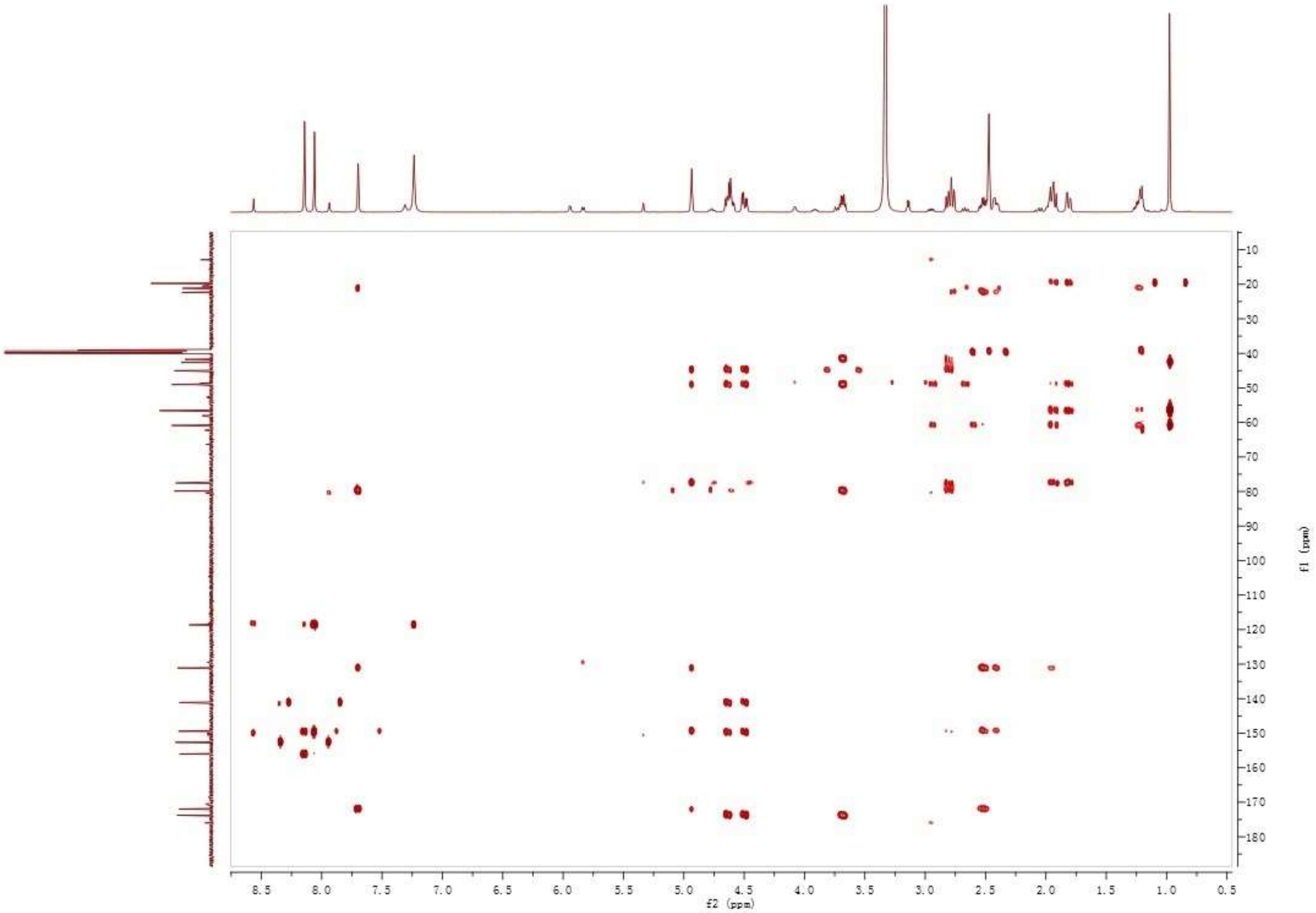

NOESY spectrum of compound **4**

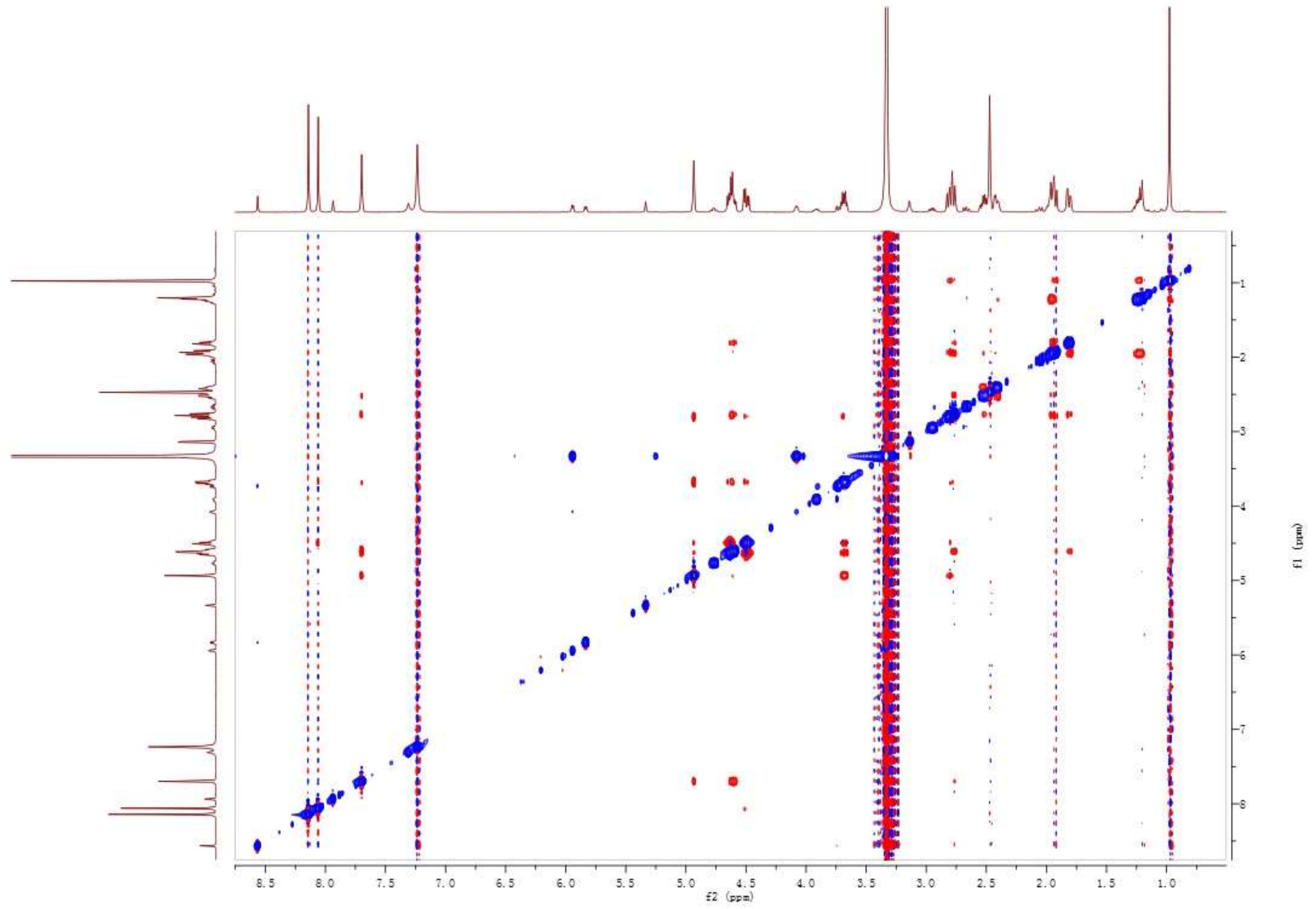

Supplement: Supplementary file 1 [file molecules-28-02119-s001.zip › molecules-2252103-SI.pdf]
